
JOURNAL INFORMATION
==============================
NLM Title Abbreviation: Biomater Investig Dent
Journal ID: Biomater Investig Dent
NLM Journal ID: 101755000
Journal ID: IABO
Journal ID: iabo21

Biomaterial Investigations in Dentistry

EISSN: 2641-5275
Publisher: MJS Publishing

ARTICLE INFORMATION
==============================
PMCID: PMC7067167
DOI: 10.1080/26415275.2019.1691842
Article ID: 1691842
Article version: 1
Subjects: Abstracts

Abstracts from the 25th European Dental Materials Conference, EDMC 2019, August 28–30, 2019, Brussels, Belgium

Publication date: 2019
Electronic publication date: 2020 Jan 6
Volume: 6
Issue: Suppl 1
Issue title: Abstracts from the 25th European Dental Materials Conference
First page: 1
Last page: 22
Copyright: © 2019 The Author(s). Published by Informa UK Limited, trading as Taylor & Francis Group.
Copyright year: 2019
Copyright holder: The Author(s)
License: This is an Open Access article distributed under the terms of the Creative Commons Attribution License (http://creativecommons.org/licenses/by/4.0/), which permits unrestricted use, distribution, and reproduction in any medium, provided the original work is properly cited.
License URL: https://creativecommons.org/licenses/by/4.0/

Page count: 22

==============================
BIOMATERIAL INVESTIGATIONS IN DENTISTRY
2019, VOL. 6, NO. S1, 1–22
https://doi.org/10.1080/26415275.2019.1691842

Taylor & Francis
Taylor & Francis Group

ABSTRACTS

OPEN ACCESS Check for updates

### Abstracts from the 25th European Dental Materials Conference, EDMC 2019, August 28–30, 2019, Brussels, Belgium

### Lithium-substituted bioactive glasses for tooth repair

A. Alaohali
King's College London, UK

**Purpose:** The Wnt canonical signaling pathway plays a crucial role in tooth regeneration, and can be upregulated by inhibition of glycogen synthase kinase 3 (GSK3). Lithium is an antagonist of (GSK3) and can be substituted in the bioactive glasses (BG), which can be used clinically to enhance the tooth repair.
**Materials and Methods:** We developed borate (LiBBG) and phosphate (LIPBG) BG which contain lithium and evaluated ion release and toxicity of their dissolution ions.
**Results:** We found that both LiPBG and LiBBG can release high levels of Li that can elevate Wnt canonical signaling, however the levels of phosphate and boron are toxic to cell cultures and limit the utility of BG in the tooth.
**Conclusion:** These data suggest that lithium-substituted BG can release therapeutic level of lithium which upregulate Wnt signaling. However, LiPBG and LiBBG are toxic to pulp cells in vitro. Nevertheless, it may be possible to use BG in vivo in non-exposed pulp tooth models that limit contact with the pulp cells.

### Fracture toughness evaluation of resin composites after environmental challenge

H. A. Algamaiah[a,b,c], J. A. Banas[a], S. R. Armstrong[a], A. Jain[a], A. Danso[d], R. Rawls[d] and E. C. Teixeira[a]
[a]The University of Iowa, College of Dentistry, Iowa City, IA; [b]King Saud University, College of Dentistry, Riyadh, SA; [c]The University of Manchester, Division of Dentistry, Manchester, UK, [d]University of Texas Health Science Center at San Antonio, TX

**Purpose:** To measure the fracture toughness of a conventional Bis-GMA-based resin composite (Filtek Supreme), experimental oxirane/acrylate-based resin composite (OASys), and ormocer-based resin composite (Admira) at baseline (control) and after a 15-day exposure to a *Streptococcus mutans* based biofilm or 30-day storage in water.
**Materials and Methods:** A 25 × 5 × 2.8 mm stainless-steel mold with 2.5 mm single edge notch at the center was used, following ASTM standards [E399-90], to fabricate 135 specimens (n = 15) based on composite material and aging conditions. For the baseline group, specimens were fabricated and then tested after 24 hours storage in water. For the biofilm challenge, specimens were randomly placed in a six-well tissue culture plate and kept at 37 °C with bacterial growth media (Brain Heart Infusion (BHI); *Streptococcus mutans)* changed daily for 15 days. BHI medium was supplemented with 0.5% sucrose to promote the establishment of a biofilm for the initial 24 hours. For the water storage challenge, specimens were kept for 30 days in 5 ml of deionized autoclaved water at 37 °C changed weekly. Fracture toughness ($K_{Ic}$) testing was carried out using a universal test with a load cell capacity of 500 N using three-point bending with 20 mm spam at a cross-head speed of 0.5 mm/min.
**Results:** Two-way ANOVA showed statistically significant differences for the interaction between materials and challenges ($p < .05$). Subsequent analysis showed that the Filtek and Admira mean baseline toughness was significantly higher than that observed for water and biofilm challenges. OASys mean toughness values in water were significantly higher than that of baseline. Toughness values for OASys in biofilm were not statistically different when compared to either water or baseline.
**Conclusion:** The fracture toughness of the commercially available composites were negatively affected by the environmental challenges of 15 days biofilm and by 30 days water storage. In contrast, the experimental composite (OASys) did not demonstrate this degradation pattern.

### Effect of filler on mechanical properties of heat-cured denture base

A. Alhotan, N. Silikas and J. Yates
School of Dentistry, The University of Manchester, UK

**Aim:** This research evaluated the surface hardness and flexural strength of heat-cured PMMA modified by the addition of three different fillers, $ZrO_2$, $TiO_2$ nanoparticles, and E-glass fibre, at various concentrations.

© 2019 The Author(s). Published by Informa UK Limited, trading as Taylor & Francis Group.
This is an Open Access article distributed under the terms of the Creative Commons Attribution License (http://creativecommons.org/licenses/by/4.0/), which permits unrestricted use, distribution, and reproduction in any medium, provided the original work is properly cited.

**Materials and Methods:** The surfaces of the fillers were modified using a silane coupling agent (y-MPS) before mixing with PMMA. In total, 130 rectangular specimens (64 × 10 × 3.3mm) were fabricated for the purpose of testing both surface hardness and flexural strength. The specimens were then divided into five groups (n = 10). Group A was the control group (unreinforced), and the remaining groups, B, C, D, and E, were reinforced with the three filler materials at concentrations of 1.5%, 3%, 5%, and 7% respectively. At different storage conditions, the surface hardness was measured using the Vickers micro-hardness test and three-point bending was performed to determine flexural strength. One-way ANOVA followed by Tukey post-hoc test were employed to compare the means across groups with a statistical difference of $p \leq .05$.
**Results:** All filled groups (B-E) had significantly higher Vickers hardness than the control group (A). Group E exhibited the highest mean hardness, while Group B had the lowest ($p < .05$). There was also a significant difference in flexural strength between the groups that contained fillers (B-E) and Group A ($p < .05$). However, there was no significant difference in flexural strength between Group E specimens, which were filled with $ZrO_2$ (89.4MPa) and $TiO_2$ (92.9MPa), and those in Group A (85.5MPa; $p > .05$).
**Conclusion:** The findings indicate that all groups consisting of silanized fillers/PMMA composites enhanced the surface hardness and flexural strength of the material. However, PMMA resin should incorporate less than 7% nanoparticles. When this ratio was exceeded, the flexural strength of the PMMA declined in comparison to alternative nanoparticle concentrations. When the content of the fillers increased, the surface hardness of the PMMA/filler composites also increased.

### Structure, property, performance relationships of potential ion-leachable resin composites

A. Almokhatieb[a,b], X. Chen[a] and D. C. Watts[a]
[a]School of Medical Sciences, The University of Manchester, UK; [b]School of Dentistry, Prince Sattam University, KSA

**Purpose:** Bioactive fillers need to be ion-leachable rather than inert. The aim of this study was to investigate the effect of water aging and unsilanated filler load on a series of experimental resin composites (RC) incorporating ion-leachable glass (ILG) filler-particles and silanated barium-borosilicate (BBS).
**Materials and Methods:** SEM, XRD and XRF were used to characterize 3 unsilanated ILG: 45S5 bioactive glass (*BG*), and two *strontium fluoro-alumino-silicate* powders (from Kent Dental) that correspond to the reactive glass compositions of FUJI IX® (*F9*) and FUJI IX EXTRA® (*F9X*). 13 RC groups were formulated with 50:50 wt% BisGMA:TEGDMA plus photoinitiator: CQ (0.5)+DMAEMA (0.8) wt%. The composites had a constant filler fraction of 72 wt%, comprised of BBS plus progressive ILG substitutions of 10, 15, 20 and 25 wt%. Water sorption/solubility were measured over 6 months. Flexural strength (FS) was measured after dry and water storage (n = 2 × 10) over 1, 7 and 30 days. For hardness (VHN), disc specimens were stored dry or in water for 24 h. (n = 6/group).
**Results:** FS and VHN significantly reduced for BG groups when aged in water especially as BG content increased. Greater ILG proportions had an increasing impact on water sorption/solubility ($p < .05$). Dry conditions had no influence on non-silanized ILG.
**Conclusion:** Ion-leaching is associated with higher water uptake and tends to impair mechanical properties of RCs. The current ISO 4049 standard for resin-composites should be revised to include evaluation of the effects of ion-release on resin-composite properties.

### Flexural strength and fibre orientation of fibre reinforced model composites

A. Alshabib, D. C. Watts and N. Silikas
University of Manchester, UK

**Purpose:** To investigate fibre orientation and flexural strength (FS) of model fibre-reinforced composites with different ratios of Bis-GMA-UDMA/Bis-EMA.
**Materials and Methods:** Five experimental fibre-reinforced resin composites were evaluated A, B, C, D and E (Table 1). The photoinitiator was CQ/amine. 60 wt% silanated barium borosilicate glass (Ø 0.7 μm) and 10 wt% E-glass fibres (3 mm and Ø15 μm) were added so that the weight percentage ratio of monomer: filler was 30:70 for all composites. FS specimens were fabricated (in a 2 × 2 × 25 mm) mould and cured using a LED source of average irradiance 1.2 W/cm$^2$ for a total of 120 s. Specimens (n = 6/group) were stored in water for 1 d, 7 d and 30 d at 37 ± 1 °C, then subjected to 3-point flexure. To determine fibre orientations, specimens from groups B, C and D were fabricated within a Teflon mould (3x6x34 mm). Fibre orientations were determined by micro computed tomography (μCT). Data were analysed by one-way ANOVA, Bonferroni post hoc test at 5% level of significance.

Table 1. Group monomer composition wt%, and flexural strength mean (MPa) at different storage intervals.

| Group/ Monomers ratios wt% | 1 D | 7 D | 30 D |
|---|---|---|---|
| A (60 G, 30 T,10 P) | 168.36 (13.4)$^{a,1}$ | 154.83 (17.6)$^{a,1}$ | 120.25 (22.1)$^{a,2}$ |
| B (50 G, 38 M,12 U) | 190.52 (22.3)$^{a,1}$ | 185.86 (27.4)$^{a,b,1}$ | 155.28 (18.1)$^{a,1}$ |
| C (50 G,30 M, 20 U) | 179.85 (23.3)$^{a,1}$ | 182.24 (26.0)$^{a,b,1}$ | 149.11 (16.9)$^{a,1}$ |
| D (50 G, 25 M, 25 U) | 179.45 (17.3)$^{a,1}$ | 145.89 (14.8)$^{a,c,1}$ | 136.79 (9.4)$^{a,2}$ |
| E (50 G, 20M, 30 U) | 180.27 (17.0)$^{a,1}$ | 146.38 (20.8)$^{a,c,1}$ | 134.11 (32.5)$^{a,2}$ |

G = bis-GMA, T = TEGDMA, P = PMMA, M = bis-EMA, U = UDMA.

**Results:** μCT showed a random alignment of fibres within the composites. Within each material, FS decreased significantly in Groups A, D and E after 30 d. Group A showed the highest reduction in FS after 30 d water storage (reduction of 28.5%). Group B had the highest FS before and after ageing, however at 30 d storage, no significant differences were seen in FS between all tested materials ($p > .05$).
**Conclusion:** Deterioration of mechanical properties of tested materials after water storage was influenced by the monomer formulation of the fibre reinforced resin composites.

### A novel self-healing dental resin composite: synthesis, characterization, and properties

K. Althaqafi, J. Satterthwaite and N. Silikas
Division of Dentistry, School of Medical Sciences, University of Manchester, UK

**Purpose:** The aims were: i) synthesis and characterization of TEGDMA-DHEPT microcapsules; ii) preparation of three self-healing dental composites (SHDC), that include varying content of microcapsules and silica dioxide ($SiO_2$); iii) evaluation of their mechanical properties.
**Materials and Methods:** Microcapsules were prepared by *in situ* polymerization of PUF shells in an oil-in-water emulsion. Optical microscope and SEM were used to measure the diameter and to observe the morphology of PUF microcapsules. FT-IR evaluated microcapsules + catalyst polymerization independently. SHDC included: Bis-GMA:TEGDMA (1:1), 1 wt% BAPO, and 0.5 wt% BPO catalyst. Inorganic phase contained 20 wt% $SiO_2$ (15 nm) and (0, 5, 10 wt%) of microcapsules. Vickers microhardness, DC, light transmission, flexural strength (FS) and elastic modulus of SHDC were measured.
**Results:** Average diameter of TEGDMA-DHEPT microcapsules ranged from 150-300 µm. SEM of the capsular shell revealed a smooth outer surface with deposits of PUF nanoparticles that facilitates resin matrix retention to the microcapsules upon composite fracture. An average shell thickness of ≤350 nm was observed. FT-IR showed that microcapsules with catalyst at 0.5 mm and 2 mm thickness had DC of 60.3% and 34.8% respectively. The DC of composites ranged from 65-67.8% immediately post-curing and 72.6-75.8% 24h after polymerization. Microhardness ranged from 21.8-25.7 VHN but it was not significant ($p > .01$). FS was adversely affected with increasing microcapsules ($p < .01$) ranging from 55 ± 4 MPa at 10 wt% microcapsules to 80 ± 12 MPa at 0 wt% microcapsules. Elastic modulus and light transmission differences were not significant.
**Conclusion:** Microcapsules were synthesized and incorporated into dental composites. Only FS was decreased by increasing microcapsules content. All other measured properties were not affected.

### Stress distribution of partially-veneered (semi-monolithic) zirconia fixed dental prostheses

F. Bakitian[a,b] and P. V. von Steyern[a]
[a]Department of Materials Science and Technology, Faculty of Odontology, Malmö University, Malmö, Sweden; [b]Department of Restorative Dentistry, Faculty of Dentistry, Umm Al-Qura University, Makkah, Saudi Arabia

**Purpose:** To evaluate influence of framework designs on stress distribution within tooth-supported partially veneered fixed dental protheses (FDPs) made of translucent zirconia under simulated loads using a 3-dimensional finite element analysis (3D FEA).
**Materials and Methods:** For linear FEA, simplified 3D solid models of prepared abutment teeth with different 3-unit FDPs based on designs were created. Five designs—monolithic (control); partially-veneered (semi-monolithic) with 0.3 mm veneer thickness (A); semimonolithic with 0.5 mm veneer thickness (B); semi-monolithic with 0.5 mm veneer thickness supported with cap design (C), and semi-monolithic with 0.5 mm veneer thickness supported with wave design (D)—were analyzed using FEA. Elastic properties of the components (bone, dentine, cement, translucent zirconia, and veneering porcelain) were gained from standard references for FEA. Simulated static forces (300 N) were applied at oblique direction over occlusal surfaces. Maximum principal stress and shear stress were calculated and analyzed among the different models.
**Results:** Model C showed lowest maximum principal stress levels in veneering porcelain compared to models A, B, and D. In zirconia framework of model C, however, maximum principal compressive stress levels were higher compared to the other models. Model A had lower maximum principal stress levels in veneering porcelain compared to model B. Maximum principal stress levels were located in veneer component of models A, B, D whereas were observed at cervical area of zirconia framework of model C. Model A had highest maximum shear stress levels while model D had lowest shear values.
**Conclusion:** Framework designs play a significant role in stress distribution of partially veneered zirconia FDPs under loading. The FDP with cap design minimizes maximum principal stress in the 0.5 mm-veneering porcelain. The FDP with a 0.3 mm-veneering porcelain has low maximum principal tensile stress in veneering porcelain but with high maximum shear stress at zirconia-veneer interface. The FDP with wave design minimizes maximum shear stress at the zirconia-veneer interface.

### Crystallography and translucency of multilayer monolithic zirconia ceramics

S. M. Cokic, F. Zhang, B. Van Meerbeek and J. Vleugels

**Purpose:** To evaluate the microstructure, optical properties and pigment characterization of highly translucent multilayer monolithic zirconia ceramics.
**Materials and Methods:** Four pre-sintered multilayer monolithic zirconia disks (Katana UTML, Katana STML, Katana ML, all from Kuraray Noritake; Lava Esthetic, 3M Oral Care) were cut in plate-shaped specimens (12 × 12 × 2mm), sintered according to the manufacturer's instructions and mirror polished. The zirconia-phase composition was characterized using X-ray diffraction (XRD) with the grain size measured using scanning electron microscopy (SEM). The translucency parameter (TP) was determined with a spectrophotometer (SpectroShade$^{TM}$ MICRO, MHT Optic Research) (n = 6/group), while pigments were characterized by X-ray fluorescence (XRF) and nanoSEM (FEI-Nova Nanosem 450, FEI) in combination with energy-dispersive X-ray spectroscopy (EDS). Statistical analysis involved one-way ANOVA and post-hoc Tukey's HSD test (α = 0.05).
**Results:** XRD confirmed that all zirconia grades investigated were partially stabilized zirconia (PSZ) with a $Y_2O_3$ content in the tetragonal $ZrO_2$ phase ranging from 2.3 to 5.4 mol% and a cubic $ZrO_2$ phase ranging from 42 to 55 wt%. Katana UTML

was most translucent (TP of 24.9 ± 0.7), followed by Lava Esthetic (23.9 ± 1.0) and Katana ML (23.4 ± 0.5); Katana STML revealed the lowest TP (22.3 ± 0.7). Optical microscopy of pre-sintered Lava Esthetic revealed dark areas in the form of agglomerates. Chemical elemental mapping using nanoSEM/EDS disclosed lanthanide elements like Erbium (Er), Praseodymium (Pr) and Terbium (Tb) in these agglomerates.
**Conclusion:** The optical properties of highly translucent multilayer monolithic zirconia ceramics underscore those of lithiumdisilicate glass-ceramics (TP of 43.4 ± 2.5, as measured earlier for e.max CAD, Ivoclar Vivadent). Rare-earth oxide-containing zirconia agglomerates were added as colour pigments to the multilayered monolithic zirconia Lava Esthetic.

### Microstructure and mechanical behavior of lithium-disilicate crystallized by microwave energy

A. B. G. de Carvalho[a], N. C. Ramos[a], J. N. Luz[b], R. M. Melo[a] and G. S. F. A. Saavedra[a]

[a]Department of Dental Materials and Prosthodontics, Institute of Science and Technology of Sao Jose dos Campos, Sao Paulo State University (UNESP); [b]Department of Dentistry, Guarulhos University (UnG)

**Purpose:** The aim of this study was to verify the viability of an alternative process of crystallization using microwave energy and to establish a crystallization protocol for a lithium disilicate ceramic; to evaluate the effect of this crystallization on microstructure and biaxial flexural strength of this glass-ceramic; and to analyze its optical properties (translucency).
**Materials and Methods:** Discs were made from blocks of lithium disilicate (IPS e.max CAD, Ivoclar Vivadent); according to ISO 6872 (dimensions: 12 × 1.2 mm). SiC papers with granulation #400, 600, 1200 and 2500 were used for specimens polishing. Half of the specimens were crystallized in a conventional furnace (C), according to the manufacturer's recommendations (840 °C; 7 minutes), and the other half were crystallized by microwave energy (MW) using two different protocols (700 °C and 850 °C; 10 minutes). The microstructural characterization was made by translucency measurements, scanning electron microscopy (SEM) and X-ray diffraction (XRD).
**Results:** The mechanical behavior was measured with the biaxial flexural test. The protocol of 700 °C for 10 minutes was not sufficient to crystallize the samples. The protocol of 850 °C for 10 minutes crystallized the samples, being the translucency parameter similar for groups C and MW. SEM and XRD analyses showed similar microstructure and crystalline patterns for both groups. The biaxial flexural strength was superior to MW group.
**Conclusion:** Microwave energy is a viable alternative to the crystallization process of lithium disilicate, resulting in a more resistant glass-ceramic and the same optical properties.

### Acknowledgments

Funding was provided by FAPESP (09577-2).

### Ceramic veneers resistance under different cementation conditions

R. R. D. G. Pignataro, L. P. C. Contreras, K. B. S. Fróis, S. Lima da Silva, L. C. C. Boaro and L. C. Anami

**Purpose:** The purpose of this research was to evaluate the flexural strength of feldspathic ceramic in thin thickness due to the cementation agent used. Discs (0.7 mm) of Vita Mark II (Vita Zahnfabrik) were polished and one of the surfaces was treated with hydrofluoric acid, silane and adhesive. The disks were randomly allocated into three groups (n = 20), according to the cement agent used: R-resin cement (Allcem Veneer); F-resin flow (Opallis Flow); C-composite resin (Opallis) previously heated at 60 °C for 60 sec. After application of the resin cement on the treated ceramic surface, an acetate film was positioned and the cement film was standardized with a micrometer (100 μm). After photo activation, the specimens remained in distilled water at 37 °C for 24 h and then were tested in biaxial flexure (1.0 mm/min) with the resin cement layer on the side submitted to tensile stresses. The flexural strength data were analyzed by 1-way ANOVA and Tukey's Test, and Weibull's analysis (a = 5%). The fractured surfaces were analyzed in stereomicroscope and scanning electron microscopy. The cementation agent influenced the flexural strength of the ceramic (p = 0.000) and lower values were observed for C (79.51 ± 6.55 MPa); R (89.46 ± 10.37 MPa) and F (87.28 ± 7.83 MPa) were superior and similar. There were overlapping confidence intervals at 95% of the Weibull modules indicating similarity between the experimental groups (R: 10.5; F: 14.3; C: 13.8), but the characteristic strength was higher for R (93.8) than for C (82.5), while F (90.6) was similar to both. The fractures originated from internal defects and from the surface submitted to tensile stress during the test. Thus, it was concluded that the flow resin may be a cementing agent that resembles resin cement with respect to the fracture strength of feldspathic ceramics.

### Reactivity and bonding capacity of a self-etch silane primer

M. Dimitriadi, M. Zafiropoulou, S. Zinelis and G. Eliades

Department of Biomaterials, School of Dentistry, National and Kapodistrian University of Athens, Greece

**Purpose:** The aim of the study was to evaluate the stability, reactivity and bond strength with a glass-ceramic (IPS Empress Press) of a self-etch silane primer (Monobond Etch & Prime/ME) based on tetrabutylammonium dihydrogen trifluoride etchant (TADF), γ-methacryloxypropyl trimethoxysilane (γ-MPTS) and bis(triethoxysilyl)ethane silane (BTSE).
**Materials and Methods:** The stability was evaluated by $^{1}H$-,$^{13}C$-,$^{31}P$-NMR spectroscopy before and after aging (sealed vials 4w/48 °C), the reactivity of fresh material was monitored by ATR-FTIR spectroscopy on Ge surfaces (0, 1, 24h storage, 50% RH/37 °C). The effect of ME on ceramic roughness *vs* 5% HF acid-etching (HF) was assessed by optical profilometry and the shear bond strength (SBS) of a flowable composite bonded to polished ceramic surfaces treated with ME (a) and HF + ME (b) were evaluated after storage in water (A:1w/37 °C and B:24h/100 °C, n = 20/material). A pure prehydrolized $\gamma$-MPTS primer (Calibra/SP) and no silane primer (NS) were used as controls for (b).
**Results:** Silanol groups (0.6-0.4 ppm/$^{1}$H) were traceable in ME, in partially condensed phase (10.1 ppm/$^{13}$C) before/after storage, but with differences in $^{31}$P peaks, indicating the presence of more P-phases than of a phosphate ester, with a phosphate-ester/TADF ratio = 1/1.3. Silanols were reactive forming a siloxane peak (1130 cm$^{-1}$) at 1h, which became stronger at 24h. HF induced significantly higher values from ME in all the roughness parameters tested (Sa, Sz, Sdr, Sc, t-test $p < .05$). For SBS, the statistical ranking of the Weibull $\sigma_0$ parameter (characteristic life) was: SP + HF, ME + HF > NS + HF, ME (A) and ME + HF, SP + HF > NS + HF > ME (B), whereas of Weibull $\beta$ parameter (reliability) was: SP + HF > ME, ME + HF, NS + HF (A) and ME + HF, SP + HF, ME > NS + HF (B) at $p < .05$. From the silane treatments, ME demonstrated the highest incidence of adhesive failures (A:40%, B:100%).
**Conclusion:** Although ME was quite stable and reactive, it demonstrated inferior strength without HF, lower than the non-silane etched control. When used with HF, ME provided the highest strength and reliability, even under aggressive storage conditions.

### Aging behaviour of novel, high-translucent CAD/CAM resin composites

V. M. Ducke and N. Ilie

Department of Operative Dentistry and Periodontology, University Hospital, Ludwig-Maximilians-University, Munich, Germany

**Purpose:** The aim of this study was to investigate the influence of artificial aging on the mechanical behaviour and fracture patterns of novel CAD/CAM resin composites (RBC).
**Materials and Methods:** Prefabricated blocks of seven materials (Voco, Grandio Blocs, GB; Ivoclar Vivadent, Tetric®CAD, TC; DMG, Luxacam Composite, LC; Shofu Blocs HC, SF; 3M, Lava™ Ultimate, LU; GC, Cerasmart®, CS; Coltene, BRILLIANT Crios, CB) were sectioned into 420 bar specimens (2 mm $\times$2 mm $\times$16 mm) and ground parallel. Flexural strength and flexural modulus were assessed in a 3-point-bending test after an aging process consisting of three sequential steps (storage in artificial saliva for 14 days, 10,000 thermo-cycles between 5/55 °C, storage in ethanol 75% for 48 hours). Fracture origins were determined in a fractographic examination using reflected light stereomicroscopy combined with transillumination and scanning electron microscopy. Effects of aging were statistically analysed using a multivariate ANOVA with Tukey's HSD post hoc test ($\alpha = 0.05$) and Weibull statistics.
**Results:** The strongest influence on the mechanical properties was exerted by the material ($p < .001$, $\eta_P^2$=0.957; $\eta_P^2$=0.770), but the influence of in vitro aging was also significant ($p < .001$, $\eta_P^2$=0.623; $\eta_P^2$=0.407). GB was the strongest and stiffest material while LC and SF showed the lowest flexural strength and CS the lowest flexural modulus ($p < .05$) in non-aged materials. Storage in artificial saliva decreased the flexural strength of all materials, apart from CS, significantly ($p < .05$). The Weibull moduli varied from 5.74 to 22.44. Fractography revealed brittle fracture features like compression curls, branching, hackle lines and fracture mirrors. The SEM analysis identified pores, agglomerates and inclusions as main fracture origins.
**Conclusions:** Storage in artificial saliva significantly reduced the mechanical properties while thermo-cycling caused only a slightly reduction in flexural modulus. Fractographic analysis revealed a significant accumulation of fractures originated inside the material, as a result of inherent flaws, after storage in alcohol.

### Bonding properties of third generation zirconia materials

M. Eldafrawy[a], S. Bekaert[a,b], M. Sadoun[c] and A. Mainjot[a,b]

[a]Dental Biomaterials Research Unit (d-BRU), Institute of Dentistry, University of Liège (ULiège), Liège, Belgium; [b]Department of Fixed Prosthodontics, Institute of Dentistry, University of Liège Hospital (CHU), Liège, Belgium; [c]MaJEB sprl, Liège, Belgium

**Purpose:** To use the Notchless Triangular Prism (NTP) test to compare the Interfacial Fracture Toughness (IFT) of 2 resin cements, Panavia-SA Cement Plus (SA) and Panavia-V5 + Ceramic Primer (V5) (Kuraray Noritake), and a resin-modified glass-ionomer cement (RMGIC) Fuji Plus (GC Corporation), with Katana zirconia CAD-CAM blocks of the third generation (Kuraray Noritake) for CEREC systems, after sandblasting with 50-µm $Al_2O_3$ particles at 0.5 bar and 2.5 bar and upon thermocycling aging.
**Materials and Methods:** Katana blocks were cut and polished to produce prisms of larger dimensions to compensate for the shrinkage after sintering. The prisms were then split, polished and sintered to produce 6.0 ± 0.1-mm half prisms. Ten prisms were bonded with SA, 10 with V5 (after pre-treatment with the dedicated ceramic primer) and 10 with Fuji Plus after sandblasting at 0.5 bar and the same procedure was repeated at 2.5 bar (n = 60 prisms). Prisms were thermocycled (10,000 cycles) and tested for IFT using the NTP test in a water bath at 36 °C. Moreover, random fractured samples were selected for fractography with SEM along with 2 rectangular samples after sandblasting with 0.5 bar and 2.5 bar.
**Results:** SA 2.5 bar gave the highest IFT significantly, followed by V5 2.5 bar then SA and V5 0.5 bar. All Fuji-Plus samples failed before testing. SA 2.5 bar showed the highest Weibull values. Fractography showed typical adhesive fracture patterns and SEM characterization highlighted the increased micro-roughness created by the 2.5 bar sandblasting pressure.
**Conclusion:** The IFT values for SA and V5 are high and comparable to values obtained in a previous work with gold-standard materials such as etched glass-ceramics, while RMGIC is not recommended for Cerec Katana Zirconia restorations. The higher IFT obtained with increased sandblasting pressure highlights the influence of micromechanical retention on interface performance. Panavia V5 and Panavia SA Cement

Plus are very well-adapted to Katana zirconia but further studies are needed to explain the better performance of SA.

### Effect of universal adhesives on dentin collagen triple-helix integrity

G. Eliades, K. Anastasiades and D. Papadogiannis

Department of Biomaterials, School of Dentistry, National and Kapodistrian University of Athens, Greece

**Purpose:** The aim of the present study was to evaluate the effect of mild- and strongly-acidic universal adhesives, on the integrity of the triple helix of type-I collagen of human coronal dentine.
**Materials and Methods:** Coronal dentin disks (h = 1mm) were prepared by horizontally sectioning the crowns of sound extracted third molars. The top surfaces were polished with 600 grit-size SiC paper, to form a smear-layer, rinsed with water (10s), dried with warm air (10s) and the central part of the specimen (0.3X0.4 mm) was analyzed by reflection FTIR microscopy. The same region was then subjected to treatment with a universal adhesive according to the manufacturer's instructions, rinsed off with water and acetone, air dried and spectra were taken again. Under these conditions dentin spectra pairs (treated/native) were obtained (n = 4) for the following adhesives: All-Bond Universal (AB), Adhese Universal (AD), Clearfil Universal Bond Quick (CQ), G-Premio Bond (GP), Prelude One (PR) and Scotchbond Universal (SB). A 37% phosphoric acid (PA) gel-etchant (Total Etch) was used as a control. The amide I peak band (1690-1585 $cm^{-1}$) of the spectra was curve fitted (Gauss area mode) into three major peaks. The area ratio of the 1670-1660 $cm^{-1}$ peak component (assigned to the a-helix structure) to the total area of the amide I fitted peaks was calculated before and after treatment. The post-treatment reduction (CR%) was expressed in percentage. Statistical analysis was performed by one-way ANOVA and Tukey test (a = 0.05).
**Results:** The results of CR% were (mean, sd): AB:10.5(2.6), AD:9.4(3.2), CQ:10.9(2.7), GP:16(2.6), PR:7.4(1.9), SB:12.3(2.3), PA:36.5(6.9). PA showed significant higher values from all adhesives, which formed a statistically homogeneous group.
**Conclusion:** The universal adhesives tested in the self-etch mode affected significantly less the triple helix of dentin type-I collagen than phosphoric acid. This may be an additional advantage of the selective enamel-etch over the total-etch technique.

### Effect of surface treatment and adhesive strategy on the bond strength of a universal adhesive to sclerotic dentin of non-carious cervical lesions

D. H. Floriani

**Purpose:** The study aimed to evaluate the bond strength of a resin composite to the sclerotic dentin of non-carious cervical lesions (NCCLs) using different surface treatments and adhesive strategies.
**Materials and Methods:** Thirty-six human premolar teeth with NCCLs were selected and randomly distributed into groups according to the dentin pre-treatment: C (control group, no treatment), DB (diamond bur roughening), and AA (air abrasion with aluminum oxide particles). Based on the adhesive strategy used with the universal adhesive, each group was subdivided into either T (total-etch) or S (self-etch) (n = 6). The teeth were restored with a nanofilled composite and stored in water at 37 °C for 24 h. The teeth were sectioned in micro-specimens (1 $mm^2$ interface area) and stressed in tension until failure (μTBS) in a universal testing machine (0.5 mm/min). Data were assessed by two-way ANOVA and Tukey's HSD test ($\alpha = 5\%$).
**Results:** Significant differences were found for the variables "surface treatment" and "adhesive approach" ($p < .05$). DB-T and AA-T groups showed significant differences when compared with their counterparts DB-S and AA-S ($p > .05$). The mean bond strength of C-T was not statistically different from all the other groups ($p > .05$).
**Conclusion:** Dentin roughening with diamond bur and total-etch approach were able to increase the bond strength of composite to sclerotic dentin of NCCLs.

### Optimization of a biomimetic dental restorative material using FEA

V. Fouquet[a*], L. Tapie[a], J. P. Attal[a] and A. Benoit[a]

[a]Innovative Dental Materials and Interfaces Research Unit (URB2i), Faculty of Dental Surgery, Paris Descartes University, France

**Introduction:** Enamel (E∼40 to 80 GPa) and dentine (E∼20 GPa) have very different mechanical properties. Over a few micrometers, the dentin-enamel junction (DEJ) shows a gradient of elastic modulus between 20 GPa and 80 GPa and cracks propagating from enamel are naturally stopped in areas close to the DEJ [1]. But dental tissues are commonly restored using a unique dental biomaterial with homogeneous and isotropic properties. High stresses are observed at the bottom of ceramic dental restorations and generally lead to its fracture or debonding in the 10 first years of treatment [2].
**Materials and Methods:** Finite element analysis was used to analyze stress distribution in a simplified ceramic restoration model composed of a 8 mm diameter cylinder loaded at its center by a spherical indenter with a 120 N force to simulate an average contact force in the molar region. Inspired by the DEJ, a functionally graded material (FGM) was introduced to reduce stress at the bottom of the restoration. The FGM is a multilayer ceramic material whose number of layers, width and range of elastic modulus have been tuned to optimize the stress reduction in the ceramic part. This FGM restoration is perfectly bonded on dentin with a 2-layers bonding system: an IDS (80 μm thick, E = 1 GPa) and an auto-adhesive luting composite (100 μm thick, E = 6.3 GPa).
**Results:** A technically achievable compromise model has been found for a 2mm thick restoration. The FGM is composed of four 250-μm-thick layers with a gradient of elastic modulus between 40 GPa and 70 GPa and is capped by a 1-mm-thick vitroceramic (E = 80GPa). We have virtually reduced by 42% the stress compared to a conventional vitroceramic restoration.

**Conclusion:** FGM ceramics are interesting on a clinical point of view because they make possible more lasting and less invasive treatment of dental tissue losses thanks to more resistant and thinner restorations.

### References

[1] Wang Z, et al. Mapping the mechanical gradient of human dentin-enamel-junction at different intratooth locations. Dent Mater. 2017.

[2] Pjetursson BE, et al. A systematic review of the survival and complication rates of all-ceramic and metal-ceramic reconstructions after an observation period of at least 3 years. Part I: single crowns. Clin Oral Implants Res. 2007;18(Suppl 3):73–85.

### Application of macro photography for the characterization of dental materials

F. Fuchs, S. Hahnel and A. Koenig

Department of Dental Prosthetics and Materials Science, Leipzig University

**Purpose:** The aim of the project was to investigate macro photography as a financially affordable approach for different applications in dental material characterization, including high-resolution imaging, topography analysis, color determination, and translucency measurements.

**Materials and Methods:** An “Olympus OM-D E-M 1 Mark II” with a “Canon MP-E 65mm f/2.8 1-5x” objective was used with different light sources (incident, transmitted light, crossed polarizers) and an automatic stacking unit for material and topographical investigations as well as standard illuminants (D50, D55, D65) for color analyzation. In order to investigate the performance of this cost effective analytical approach, a bracket with highly distinct surface topography, CAD/CAM composite samples with different translucencies, and tooth colors as well as corresponding thin sections were analyzed with the image software “Helicon Focus Pro” and “ImageJ”.

**Results:** The samples were visualized in 50 million pixel images with a 5:1 magnification. A small (high numbered) aperture was used to achieve a high depth of field (2.240 mm) and sharpness across a three dimensional surface of the curved samples within a single picture. For CAD/CAM composite samples, digital tooth colors and translucencies could be reproducibly analyzed. Using focus variation (automatic stacking), high magnifications and big (low numbered) apertures, the three dimensional surface of the specimen could be analyzed with a height-resolution of 48 $\mu$m within the given setup.

**Conclusion:** Macro photography with automatic stacking is a cost effective and powerful novel approach for the two- and three-dimensional visualization and analysis of curved dental material samples with distinct topography. The simple setup is modularly expandable and capable of being expanded with state-of-the-art technology.

### Effect of bioactive glass-ceramic associated to propolis on dentin-adhesive interface

R. Geng-Vivanco, R. Tonani-Torrieri, A. B. Silva, F. M. Oliveira and F. C. P. Pires-de-Souza

**Purpose:** This study evaluated the effect of bioglass solution (Biosilicate) associated with propolis on bond strength (BS) of composite restorations to dentin.

**Materials and Methods:** Human sound molars (160) were selected and prepared (5 mm x 4 mm x 4 mm) using carbide burs. Afterwards, they were separated into eight groups (n = 20) according to the treatment received before the adhesive system (Adper Single Bond Universal, 3M ESPE): Control Group – Adhesive System; CHX Group – Chlorhexidine 0.12% (CHX); Bio Group – Biosilicate solution at 10% (Bio); P16 Group – Propolis extract with low levels of polyphenols (P16); P45 Group – Propolis extract with high levels of polyphenols (P45); CHXBio Group – CHX + Bio; P16Bio Group – P16 + Bio; P45Bio Group – P45 + Bio. After restorative procedures (Filtek Z350XT, 3M ESPE), samples were sectioned into sticks in accordance with the “non-trimming” technique, separated and stored in distilled water at 37 °C for 24h, 6 months and 1 year. After that, the sticks were submitted to microtensile test (0.5mm/min). The fracture pattern was analyzed by digital microscope (VH-M100).

**Results:** According to the results (2-way ANOVA, Tukey, $p < .05$), between the groups tested after 6 months, P16 Group revealed higher bond strength ($p < .05$) compared with the control group. There was no significant difference ($p > .05$) among the other groups, regardless treatment or storage time. After 24h, samples showed more non-adhesive fractures when they were treated with propolis extract and/or Biosilicate.

**Conclusion:** It was concluded that the pre-treatments and the storage times do not negatively affect the bond strength of the composite restorations to dentin. The fracture patterns were mainly non-adhesive.

### Water sorption of polyphenylene orthodontic archwires

A. J. Goldberg

Department of Biomedical Engineering, School of Dental Medicine, University of Connecticut Health Center, CT, USA

**Purpose:** There is continuing demand for an esthetic, yet biomechanically effective orthodontic archwire. Translucent polyphenylenes may possess an appropriate combination of properties for this application. Compared to the approach of coating metal wires, these non-reinforced polymers also have potential biomechanical design benefits, but like all polymers, water sorption may affect properties. Accordingly, it is important to understand the effects of water sorption on relevant mechanical properties.

**Materials and Methods:** Polyphenylene wires with clinically relevant dimensions were extruded (Solvay, Inc.). Water sorption and tensile properties were measured in the as-received condition, after standardized drying (conditioning), after storage at 37°C in air or immersed in de-ionized water for up to 12 weeks, and after re-conditioning. Groups were compared with ANOVA.

**Results:** The immersed samples had a statistically significant weight gain relative to the conditioned controls ($p < .0001$). Typical of thermoplastics, the samples were saturated in 24 hours, and had a weight gain of 0.41 ± 0.20%. The mean water sorption over all time points was 0.50 ± 0.26%. There was no trend over time, although the one-week sample was statistically greater than the 6 and 12-week samples ($p = 0.030$). Reconditioned samples returned to their pre-immersion weight ($p > 0.05$), demonstrating that the water sorption was reversible and there were no measurable water-soluble components in the wire. The largest decrease in tensile properties occurred during the first 24 hours of immersion, when elastic modulus, yield strength and ultimate strength decreased by 5%, 8% and 5% respectively, however, neither these decreases nor the changes over the longer test periods were statistically significant ($P > .05$).

**Conclusion:** The water sorption of the polyphenylene archwires was typical for a thermoplastic polymer. No change in the tensile properties due to water sorption would be expected during typical treatment times.

### Inhomogenous curing wavelength distribution affects spatial photopolymerisation in multiple photoinitiator systems

M. Hadis, A. C. Shortall and W. M. Palin

**Purpose:** Investigate effects of multiple wavelengths (2 × blue, 470nm; 1 × violet, 405 nm) and camphorquinone (CQ) and/or monoacylphosphine oxide (TPO) on spatial degree of conversion (DC).

**Materials and Methods:** Filled-resins (35wt%/0.7μm SrF; 5wt%/14nm fumed-silica) were formulated (3:2 BisGMA/TEGDMA) containing either (a) 1xCQ/DMAEMA or (b) 1 × TPO ($1.33 \times 10^{-2}$ mol.L$^{-1}$, respectively), (c) 1 × CQ +1 × TPO, or (d) 0.5 × CQ +0.5 × TPO. Beam-profile (Bluephase-Style, Ivoclar Vivadent) was assessed with a homogenising ("new-tip", NT) and non-homogenising tip ("original-tip", OT) using a CCD camera and UV-Vis spectrometer. Disc specimens (12mm diameter/1mm thick) were cured (3s or 10s) at the upper surface. Spatial DC was assessed immediately after curing using a FTIR-microscope (Nicolet iN10 MX, Thermo-Scientific; ATR mode) in 500μm increments. The aliphatic/aromatic peak height ratio of cured specimens was normalised against uncured materials measured on a Nicolet 6700 (Thermo-Scientific).

**Results:** NT reduced irradiance (OT: 1585 ± 496 mW/cm$^2$; NT: 1180 ± 36 mW/cm$^2$; $p < .05$) albeit with better beam profile. For OT, the output wavelength was dependent on the adjacent local diode: when a 4mm sensor was placed over the violet diode (at the tip surface), the blue diodes were undetected. For NT, a 1:1 violet/visible peak was detected. Local DC was dependent on the photoinitiator(s) present and the local diode wavelength/irradiance ($p < .05$). CQ-only materials exhibited 2 regions of high DC (up-to ~30% and 80% after 3s and 10s, respectively) corresponding to the blue diodes, whereas other areas were lower (<30% after 3s and <50% after 10s). For TPO-only materials, DC was higher in the violet areas (>60% even after 3s; $p < .05$). Other areas remained uncured. Multiple initiators and beam homogenisation with NT improved spatial DC and the non-homogenous cure became less apparent at 10s compared to 3s.

**Conclusion:** Local DC is affected by photoinitiator and local diode wavelength. Beam homogenisation and multiple photoinitiators can improve spatial DC with sufficient exposure.

### Light-curable, degradable and non-cytotoxic polymer-based composite for load bearing applications

S. Hamidi[a], R. Williams[b], L. Grover[b] and W. Palin[a]
[a]School of Dentistry, University of Birmingham, Birmingham, UK; [b]Institute of Translational Medicine (ITM), Birmingham, UK

**Purpose:** To design and develop a light-curable, degradable and relatively non-cytotoxic polymer-based system that will provide stability and biomechanics necessary during the early stages of tissue regeneration processes. The novel polymer system is based on polylactide (PLA) and polycaprolactone (PCL).

**Materials and Methods:** Various composition mixtures of PLA to PCL were prepared with CQ/DMAEMA (1:2 wt %) as photoinitiator and co-initiator respectively. Real-time photo-polymerisation and degree of conversion (DC) at various curing depths (0.5, 3 & 6 mm), at 25°C and physiological temperature (37°C) were assessed using Fourier Transformed infra-red spectroscopy (FTIRS) using Spectra X light engine, which was calibrated to deliver ~1000 mW/cm$^2$ (± 0.10%). Initial micro-tensile and compressive strength tests were performed at strain rates of 0.05/s [1] and 1/s [2]. Enzymatic degradation of neat and filled (calcium phosphate) polymer systems were assessed at ten-fold and physiological concentration of lipase using DPBS, for 8 weeks.

**Results and Discussion:** Depending on the composition mixtures and curing depth, final DCs ranged from 68-95% polymerisation conversion. Change in environmental temperature was statistically significant ($p > 0.05$) with >5% increase in DC at 37 °C across all composition mixtures. At both strain rates, elastic modulus (2-18 GPa) increased in accordance with PLA content in the mixture. While the modulus was higher under tension, the ultimate strength (272-334 MPa) was significantly higher under compression. Adequate DC of 76 and 68% was achieved at each curing depth at both 5 and 10 wt% filler (un-silanated) content respectively. Enzymatic degradation indicated very slowly degradation due to the inherent hydrophobic and highly cross-linked nature of the polymer.

**Conclusion:** The novel polymer system has the ability to rapidly photo-polymerise with enhanced mechanical properties. Its slow degradation regime coupled with its strength retention may provide the necessary stability and biomechanics during the early stages of tooth and bone remineralisation as a result of calcium phosphate release through the polymer matrix.

### References

[1] Zhou and Hsiung. Depth-dependent mechanical properties of enamel by nanoindentation. J Biomed Mater Res A. 2007;81(2007):66–74.

[2] McElhaney JH. Dynamic response of bone and muscle tissue. J Appl Physiol. 1966;21:1231–1236.

### Mechanical properties of soft relining material modified with chitosan salts

M. Herla[a], H. Meissner[a], K. Boening[a] and K. Walczak[a]

[a]Carl Gustav Carus Faculty of Medicine, Technische Universität Dresden, Department of Prosthetic Dentistry, Dresden, Germany

**Purpose:** Evaluation of mechanical properties of a soft denture relining material modified with chitosan salts (CS-salts) as possible antimicrobial agents for treating and preventing denture stomatitis.
**Materials and Methods:** Chitosan salts: HCL (CS-HCl) and Glutamate (CS-G) were added to an A-silicone based soft denture relining material (Ufi Gel) at four different concentrations (0.1%, 0.2%, 0.4% and 1% by weight). Ten specimens per group (n = 10, Ø 35 mm, 6 mm height) were produced according to ISO 10139-2:2016. During the experiment, all specimens were stored in distilled water at 37 °C. Shore A hardness and surface roughness (Ra) were evaluated at four points in time: 24 h (T1), 7 (T2), 14 (T3) and 30 days (T4). Kruskal-Wallis and U-test (Bonferroni-Holm adjusted) were used for statistical analysis ($p \leq .05$).
**Results:** At T1, T2, T3 and T4 all CS-salt modified groups were significantly rougher than the control group (raw and adj. $p < .001$). Ra increased over time and with increasing concentration of CS-salts. All groups modified with CS-salts showed Ra values higher than 0.2 μm. Shore A hardness increased over time and showed the highest values at T3 for all groups. Compared to the control group all CS-G groups showed significantly increased hardness. No significant differences at T1 for CS-HCl 0.1% and 0.2% group, as well as at T4 for CS-HCl 0.1% and 1% group were shown (raw and adj. *p*). All groups fulfilled ISO 10139-2:2016 criteria for Shore A hardness.
**Conclusion:** Changes in surface roughness and Shore A hardness were observed by CS-salts modified soft relining material. Regarding Shore A hardness all specimens showed acceptable values. When modified with CS-salts surface roughness increased over the 0.2 μm threshold that can increase biofilm accumulation. Further studies to evaluate the antimicrobial potential of the CS-salts modified material by unfavourable surface roughness are needed.

### A comparative study of light transmission through various dental restorative materials and the tooth structure

N. Ilie

Department of Operative Dentistry and Periodontology, University Hospital, Ludwig-Maximilians-University, Munich, Germany

**Purpose:** The study aim to quantify and compare the amount of light that passes through different types of direct and indirect restorative materials, comprising light-cured resin composites either regular or bulk-fill, CAD/CAM restoratives such as ceramics, glass-ceramics, resin composites and PMMA resin as well as the tooth structure.
**Materials and Methods:** Individual sets (n = 6) of plane-parallel test specimens (2 mm) of 32 restorative materials and the tooth structure have been prepared. Optical properties (transmittance, T; absorbance, A; and opacity, O) of each material were calculated from the relation between incident and transmitted irradiance (I(d)), when using a violet-blue light curing unit. Incident and transmitted irradiance were assessed in real-time on a spectrophotometer. A multivariate analysis (general linear model) assessed the effects of various parameters on the optical properties.
**Results:** A very strong influence of the parameter *material* has been identified on I(d) ($p < 0.001$; partial eta squared $\eta_P^2 = 0.953$), T ($p < 0.001$; $\eta_P^2 = 0.951$), A ($p < 0.001$; $\eta_P^2 = 0.925$) and O ($p < 0.001$; $\eta_P^2 = 0.886$), while the effect of the parameter *material type* was not significant ($p = .079$, .05, .05 and .051, respectively). Light attenuation differed significantly by material within each shade category and by shade category within analysed material.
**Conclusions:** Attenuation of light through restorative materials and tooth structure is high (59.9 % to 94.9%) thus deficits in polymerisation are difficult to compensate for by additional light exposure at the end of the restorative process.

### Biomimetic toothpaste and bioactive restorative material effect against secondary caries

A. C. Ionescu[a], P. Delvecchio[b], V. Zambelli[b], G. Bellani[b], M. Belluz[a], D. Augusti[a] and E. Brambilla[a]

[a]Oral Microbiology and Biomaterials Laboratory, Department of Biomedical, Surgical, and Dental Sciences, Università degli Studi di Milano, Milan, Italy; [b]School of Medicine and Surgery, Università degli Studi di Milano Bicocca, Monza, Italy

**Purpose:** This study aimed to evaluate the remineralization ability of a biomimetic toothpaste on an extracted tooth restored with a glass-ionomer (GIC) restoration and presenting secondary caries (SC).
**Materials and Methods:** An upper right wisdom tooth was extracted due to irreversible pulpitis two years after placement of restoration using high-viscosity GIC (Equia, GC Corp, Tokyo, Japan). After written, informed consent of the donor, the tooth was treated with a remineralizing toothpaste containing citrate-stabilized amorphous calcium phosphate and 1450 ppm fluoride (Biosmalto Mousse Protezione Carie™, Curasept S.p.A., Saronno, Italy). The toothpaste was mixed with distilled water to a 1:3 slurry, then the tooth was treated with the slurry for 5 min, twice a day. Before treatment (t = 0), after 14 days, and after two months the tooth was scanned using micro-CT (Skyscan 1176, 9μm resolution, 80KV, 300mA). After micro-CT scan, the tooth was sectioned and observed using scanning electron microscope and energy-disperse X-ray spectroscopy probe (SEM-EDS).
**Results:** Micro-CT at t = 0 revealed that the HVGIC material was placed in deep caries vestibular cavity. It caused an increase in mineral density in dentin tissue close to the restoration, and a decrease in mineral density of the restoration close to dental tissues, suggesting a migration of ions towards dental tissues over time, as confirmed by EDS. Secondary caries occurred at the cervical margin of the restoration. Toothpaste treatment caused a progressive increase in mineral density of secondary caries close to the surface, closing the demineralized gap between restoration and tooth. EDS identified elements belonging to the toothpaste at the interface between restoration and tooth.
**Conclusion:** In vitro treatment with a remineralizing toothpaste was able to close the gap left by secondary caries next to a GIC restoration in vivo. Combining bioactive restoration materials and biomimetic toothpaste may be useful against secondary caries occurrence.

### Load-bearing capacity of biomimetic cusp-replacing resin composite restorations

F. Keulemans[a], L. Lassila[a], W. Jaquet[b,c,d], P. K. Vallittu[a,e] and S. Garoushi[a]

[a]Department of Biomaterials Science and Turku Clinical Biomaterials Center TCBC Institute of Dentistry, University of Turku, Turku, Finland; [b]Oral Health Research Group ORHE, Faculty of Medicine and Pharmacy, VUB Vrije Universiteit Brussel, Brussels, Belgium; [c]Department of Educational Sciences EDWE-LOCI, Faculty of Psychology and Educational Sciences, VUB Vrije Universiteit Brussel, Brussels, Belgium; [d]Department of Periodontology and Oral Implantology, Dental School, Faculty of Medicine and Health Sciences, Ghent University, Ghent, Belgium; [e]City of Turku Welfare Division, Oral Health Care, Turku, Finland

**Purpose:** The aim of this study was to evaluate the load-bearing capacity and fracture behavior of biomimetic cusp-replacing restorations made of a dentine-replacing short fiber-reinforced composite (SFRC) and an enamel-replacing particulate filler composite (PFC).
**Materials and Methods:** Four groups of posterior cusp-replacing restorations were fabricated (n = 8/group). Group A: made of PFC (G-aenial Posterior, GC Corp), Group B: made of SFRC (everX posterior, GC Corp) as substructure with a 2 mm thick surface layer of PFC (G-aenial Posterior, GC Corp), Group C: made only of SFRC (everX posterior, GC Corp), and Group D: made of indirect PFC (Lava Ultimate, 3M ESPE). Intact teeth (Group E) without any preparation were used as control. Restorations were subjected to a static loading test until fracture occurred. Failure modes were visually examined. The dataset was checked for normality and subsequently statistically analyzed by Kruskal-Wallis followed by Mann-Whitney U test ($p = 0.05$).
**Results:** Direct cusp-replacing restorations made of PFC (group B: 1861 ± 550N) and PFC in combination with SFRC (group C: 1928 ± 465N) were able to restore heavily compromised teeth up to the original fracture strength of intact teeth (group E: 1820 ± 524N). Indirect PFC restorations (group D) exhibited significant higher load-bearing capacity (2748 ± 458N) than direct PFC restorations (group B) (Mann-Whitney U = 6, $p = 0.005$) and direct biomimetic restorations (group C) (Mann-Whitney U = 6, $p = 0.005$). No statistically significant different load-bearing capacity was found between biomimetic restorations (Group B) and direct PFC restorations (Group A) (Mann-Whitney U = 30, $p = 0.878$). The strongest restorations (group D) failed in all cases due to catastrophic cusp fracture (unfavorable failure). Failure mode analysis furthermore revealed that biomimetic restorations (group C: 12.5% unfavorable failures) showed fewer unfavorable failures in comparison to direct PFC restorations (group B: 50% unfavorable failures).
**Conclusion:** Biomimetic direct restorations combining SFRC as substructure and PFC as surface layer displayed promising performance related to fracture behavior.

### *In Vivo* Low temperature degradation of monolithic zirconia restorations

V. Koenig[a,b], C. Wulfman[c], N. Dupont[a,b], S. Bekaert[a,b], S. Le Goff[c], M. Eldafrawy[a], G. Martin[a], T. Douillard[d], J. Chevalier[d], A. Vanheusden[a,b] and A. Mainjot[a,b]

[a]Dental Biomaterials Research Unit (d-BRU), University of Liège (ULiège), Belgium; [b]Department of Fixed Prosthodontics, University of Liège Hospital (CHU); [c]Unité de Recherches en Biomatériaux Innovants et Interfaces (URB2i), Université Paris Descartes, France; [d]Université de Lyon, INSA Lyon, CNRS, MATEIS, Villeurbanne, France

**Purpose:** To evaluate in vivo Low Temperature Degradation (LTD) of second-generation zirconia restorations using an original protocol including ex vivo analyses of zirconia crystalline microstructure, focusing on the effect of occlusal stress and glaze protection.
**Materials and Methods:** 75 posterior monolithic zirconia restorations (total: 101 tooth elements) (Lava Plus, 3M ESPE) were included. On each element, several areas were determined: submitted and non-submitted to mastication mechanical stress, glazed and nonglazed. Before prosthesis placement, ex vivo analyses regarding LTD and glaze wear were performed using Raman spectroscopy and SEM. Clinical evaluation and ex vivo analyses were repeated after 6 months and each year for up to 5 years.
**Results:** 95 elements including 480 areas were evaluated at 2-year follow-up. The percentage of transformed areas was 4.0% at baseline (due to occlusal grinding) and 12.1% at 2 years. The percentage of areas presenting a monoclinic volume fraction superior to 50% (considered as a threshold beyond which material properties can be affected) was 2.3%. Transformation was significantly more frequently detected on axial areas than on occlusal areas (17.9% and 8.4% respectively), these results being explained by transformed grain pullout. The survival rate of restorations was 93.3% and the success rate was 84.0%. Most complications occurred in patients with clinical signs of bruxism (61.7% of patients). Complications were also observed on antagonistic teeth. Glaze wears out from all occlusal contact areas.
**Conclusion:** LTD develops in Lava Plus zirconia after 1 year and increases linearly with time. Glaze wears out and consequently cannot protect from ageing. The hypothesis is that LTD kinetic is low and should have no impact on the lifespan of dental prostheses. The high zirconia stiffness and lack of stress resilience could be suspected to increase the failure rate, particularly in patients with clinical signs of bruxism, the weak link being the restoration support or the antagonist.

### The effect of aging on high transluscent zirconia

W. Kou[a], K. Garbrielsson[a], A. Borhani[a], M. Carlborg[b] and M. M. Thorén[a]

[a]Department of Odontology, Umeå University, Umeå, Sweden; [b]Department of Applied Physics and Electronics, Umeå University, Umeå, Sweden

Zirconia is known for its high flexural strength but lacking translucency. A new type of high translucent zirconia with 5 mol% yttria has become commercially available with a larger fraction of cubic zirconia. According to the manufacturer it has an increased translucency and retained mechanical strength.

**Purpose:** This study aims to analyse two products of high translucent zirconia, DD cubeX$^2$ and Prettau Anterior, concerning the effects of aging on surface roughness, translucency, crystal structure and biaxial flexural strength. The null hypothesis is that there is no difference between the two materials. Discs from DD cubeX$^2$ and Prettau Anterior were analysed regarding surface roughness, visible transmittance and phase transformation.

**Materials and Methods:** The measurements were performed before aging and after 5 and 10 hours of aging respectively. Also, biaxial flexure strength tests of unaged discs and the discs aged for 10 hours were made.

**Results:** In its unaged state, DD cubeX$^2$ had the highest mean biaxial flexural strength and showed a significant reduction in flexural strength after aging ($p < .05$). Prettau Anterior showed an insignificant increase in flexural strength after aging ($p > .05$).

**Conclusion:** Aging had no significant effect on translucency or surface roughness for neither of the materials ($p > .05$).

### Two-body wear of CAD/CAM restorative materials using zirconia antagonists

E. Maier, I. Knepper, A. Petschelt, R. Belli and U. Lohbauer

Dental Clinic 1 – Operative Dentistry and Periodontology, Friedrich-Alexander University Erlangen-Nuremberg, Erlangen, Germany

**Purpose:** To investigate two-body wear of different CAD/CAM and direct restorative materials, opposed to zirconia-ceramic ($ZrO_2$) antagonists in a dual-axis chewing simulator.

**Materials and Methods:** Six CAD-CAM based composite and hybrid-ceramic blocs (Grandio blocs, VOCO, Cuxhaven, Germany/Tetric CAD, Ivoclar Vivadent, Schaan, Liechtenstein/Lava Ultimate, 3M EPSE, Seefeld, Germany/Brilliant Crios, Coltene, Altstätten, Switzerland/Cerasmart, GC, Tokyo, Japan/VITA Enamic, VITA, Bad Säckingen, Germany), one lithium disilicate ceramic CAD-CAM material (IPS e.max CAD, Ivoclar Vivadent), three direct composite resins (Clearfil majesty posterior, Kuraray, Tokyo/Grandio SO, VOCO/Filtek Supreme XTE, 3M ESPE), amalgam (Silber70-Solo, DMG, Hamburg, Germany) and bovine enamel were tested in the chewing simulator. Eight flat specimens per material were produced, grinded and polished, stored at 37 °C in distilled water for four weeks and tested (49N, 37 °C, 1,200,000 cycles) in a chewing simulator (SD Mechatronik, Feldkirchen, Germany) with $ZrO_2$ antagonists (Y-TZP, 6mm diameter). Wear resistance was analysed in a 3D optical noncontact profilometer (CT100, CyberTechnologies, Eching-Dietersheim, Germany) equipped with the confocal white-light sensor CHR-600 (CyberTechnologies (res.=0.02µm)) measuring vertical substance loss and volume loss. Data were statistically analysed using one-wayANOVA and Student-Newman-Keuls posthoc-routine (p=.05).

**Results:** Bovine enamel reached the highest wear-resistance against the $ZrO_2$ antagonist. All materials under investigation were abraded, Lava Ultimate (LU, 2.722 mm$^3$) and Filtek Supreme XTE (FS, 2.848 mm$^3$) showed the significantly highest mean volume losses, followed by IPS e.max CAD (EC, 1.414mm$^3$). Vertical substance loss thereby correlated ($r^2$= 0.9921) with volume loss. Materials (direct/indirect) with analogical filler technologies showed similar wear resistance.

**Conclusion:** Except of FS the direct resin composites showed comparable high wear-resistance to amalgam and bovine enamel, while indirect materials were less abrasion-resistant to zirconia. The lithium disilicate ceramic EC performed worse than the polymer-based bloc materials (except LU). Materials with porous nano-cluster-fillers (LU, FS) showed reduced wear resistance against the $ZrO_2$ antagonist.

### Roughness of stained dental ceramics

J. D. M. Matos, M. T. V. Grabgeiro, Alcindo Bernardi Jr, A. M. O. Dal Piva, J. P. Tribst, L. C. C. Boaro and L. C. Anami

**Purpose:** The objectives of this study were to evaluate the roughness of monolithic ceramics after characterization, varying characterization techniques when possible, and to evaluate the roughness of polymer-infiltrated ceramics (PIC) by after staining, varying surface treatments prior to stain, as well as the presence of the glaze layer.

**Materials and Methods:** The monolithic ceramics - high translucency zirconia (YZHT), feldspathic ceramics (FD) and zirconia-reinforced lithium silicate (ZLS) – were stained and received glaze. For ZLS the stain was applied in a single-step during crystallization (ZLS1) or at different times and burns (ZLS2). PIC received the stain layer after polishing (P/PG), after application of hydrofluoric acid and silanization (C/CG), after blasting with 50 µm $Al_2O_3$ and silanization (J/JG) or after silanization with a self-etching agent (S/SG). Subgroups "G" also received the glaze layer. The mean surface roughness (Ra) was measured using a contact profilometer, and surfaces were also evaluated by scanning electron microscopy (SEM). After confirming the normality assumptions, the roughness data were evaluated by 1-way ANOVA (material type) for the first part of this study, or by 2-way ANOVA (surface treatment x presence of glaze) for the second stage. Differences were detected by the Tukey's test (a = 5%).

**Results:** FD showed a higher mean roughness than ZLS1/ZLS2, while YZHT was similar to all groups (p = 0.000). In the second stage, there was influence of the interaction of surface treatment factors and glaze presence (p = 0.000), with the highest mean roughness found for S group. Glazed PIC groups were all similar. The glaze factor only influenced the surface treatments J and S, and in these two the presence of glaze resulted in a less rough surface.

**Conclusion:** Among the monolithic ceramics, FD presents mean roughness superior to ZLS. For PIC the presence of glaze results in similar mean roughness regardless of the surface treatment.

### Non-silicate nanoparticles for improved nanohybrid composite resins

L. Nakanishi[a], M. R. Kaizer[b], S. Brandeburski[c], S. S. Cava[a], A. Della Bona[c], Y. Zhang[d] and R. R. Moraes[a]

[a]Federal University of Pelotas, Brazil; [b]Positivo University, Brazil; [c]University of Passo Fundo, Brazil; [d]New York University, USA

**Purpose:** The aim of this study was to coat the surface of zirconia and alumina nanoparticles with a silica-rich layer, use the particles to prepare experimental nanohybrid composite resins, and characterize the composites.
**Materials and Methods:** Bimodal composites were prepared containing 60 wt% silanated barium borosilicate particles and similar volume fractions of silica-coated zirconia (ZrSi) or silica-coated alumina (AlSi). Silica-based nanoparticles (Aerosil OX50 and Aerosil 150) of similar sizes to ZrSi and AlSi particles were tested as references. The nanofill composite resin Filtek Z350 (3M ESPE) served as control. All particles were silanated (5 wt%) and loaded to a photoactivated Bis-GMA/UDMA/Bis-EMA/TEGDMA comonomer matrix. Characterization of the composites involved analyses of viscosity, surface topography, degree of C=C conversion, depth of cure, opacity, radio-opacity, Knoop hardness, and edge strength. Flexural strength and modulus, and fracture toughness ($K_IC$) were measured immediately and after aging the specimens using 15,000 thermal cycles. Data were analyzed at $p < .05$.
**Results:** The composites showed thixotropic behavior. ZrSi and AlSi particles were not as well dispersed in the comonomer matrix as silica particles were. C=C conversion, depth of cure, and opacity were slightly reduced by incorporation of ZrSi, although radio-opacity and hardness were increased. Composites with ZrSi and AlSi particles showed no alteration in flexural strength and modulus from before to after aging, whereas silica-based composites and the control showed reductions up to 17%. Aging resulted in less than 18% reduction in fracture toughness for composites containing ZrSi or AlSi contrasted to reductions between 24% and 42% for the reference and control materials. Edge strength was significantly higher when AlSi particles were used.
**Conclusion:** The silica-coated zirconia and alumina nanoparticles were effective silanated and generated nanohybrid composite resins with improved and more stable mechanical properties as compared with the experimental silica-based nanohybrids and the proprietary nanofill composite resin tested.

### Zirconia-reinforced lithium silicates: clinical performance of a novel ceramic material

H. Nassar

Faculty of Oral and Dental Medicine, Cairo University, Cairo, Egypt

The newly introduced zirconia-reinforced lithium silicate ceramic material is suggested in many in-vitro studies to exhibit high flexural strength, fracture toughness and superior machinability when compared to other hybrid and glass-based ceramic materials without compromising the translucency.
**Purpose:** The purpose of these two clinical case reports is to evaluate the clinical performance and complications rate of zirconia-reinforced lithium silicate posterior partial crowns.
**Materials and Methods:** Two clinical cases with a total of 4 posterior teeth (2 premolar and 2 molars) were restored with zirconia-reinforced lithium silicate posterior partial crowns. After teeth preparation, partial crowns were CAD/CAM laboratory fabricated and adhesively luted with dual-polymerizing resin cement. Clinical evaluation of partial crowns was performed according to the Modified United States Public Health Service (USPHS) at baseline, 3, 6, and 12 months post-insertion. Deviations in color match were assessed both visually and digitally using spectrophotometer Vita Easyshade® V
**Results:** All restorations remained in situ in good function after 12 months observation period. Bravo ratings were reported for marginal discoloration and color matching in one restoration after 12 months.
**Conclusion:** Zirconia-reinforced lithium silicate partial crowns present a reliable treatment modality and a satisfactory clinical alternative to complete coverage crowns for restoring large defects in posterior teeth. Specific guidelines regarding color characterization, preparation design and material thickness should be followed for a successful restoration. Due to limited clinical evidence regarding this novel material, a careful case selection should be applied.

### The effect of resin infiltration on the dental surface and SEM

L. Nehme, G. Najjar, R. Habchi and M. Daou

Faculty of Dentistry USJ, Saint Joseph University

**Introduction:** Initial caries (white spots), fluorosis, traumatic hypomineralization and incisive molar hypomineralization (MIH) all have clinical symptoms involving white lesions of the enamel. There are several techniques for treating these spots among which may be mentioned: the application of remineralization agents (CPP-ACP, CPP-ACPF, fluorine), microabrasion, composite lamination. Currently there is a new micro-invasive technique for the masking of these spots called “resin infiltration”. Icon® (DMG) is the only commercialized product that meets the infiltration principle. The purpose of this study is to prove the aesthetic effect of resin infiltration (Icon®) on white spots and to show its effect on surface condition.
**Materials and Methods:** The study is divided into 2 parts: in vivo and in vitro. The in vivo part consists of 5 clinical cases with white lesions in the central incisors. Patients are treated by resin infiltration Icon® (DMG) according to the protocol established by the manufacturer. The in vitro part consists of a milk molar extracted with a white spot at its proximal surface. The lesion is divided in two: one part was subjected to the same treatment and the other part remained intact. Then the surface condition at the infiltrated white spot was compared to that of the intact lesion and vestibular surface using the SEM.
**Results:** Among the 5 clinical cases in vivo, only 2 cases failed, whereas in the 3 other clinical cases, the patients were satisfied with the result obtained from an aesthetic point of view and improvement of the surface condition. According to the in vitro study by SEM, it turned out that the Icon® makes it possible to obtain a less rough surface state, although it is not exactly like that of the healthy enamel.

**Conclusion and discussion:** We had an improvement especially in the case of white spot of carious and traumatic origin whereas the problem lies in the cases of MIH or the lesions are deeper requiring a milling or microabrasion or the application several times of the stage of etching (the icon-etch). This results in a slight loss of substance forming a concavity which will be filled by composite.

### One-Step No-prep treatment of worn dentition using PICNs

J. Oudkerk[a,b], M. Eldafrawy[b], S. Bekaert[a,b], C. Grenade[a,b], A. Vanheusden[a,b] and A. Mainjot[a,b]

[a]Department of Fixed Prosthodontics, Institute of Dentistry, University of Liège Hospital (CHU), Liège, Belgium; [b]Dental Biomaterials Research Unit (d-BRU), Institute of Dentistry, University of Liège, (ULiège), Liège, Belgium

**Purpose:** To evaluate the One-step No-prep treatment of worn dentition, which is a minimally invasive approach without provisional phase and using Polymer Infiltrated Ceramic Network (PICN) CAD-CAM composite restorations (Vita Enamic).

**Materials and Methods:** Seven patients with severe tooth wear (BEWE score >13), a maximum of two missing teeth and a significant VDO loss were included. Etiology of tooth wear was determined by questionnaire and diagnosis of temporo-mandibular disorders (TMD) was performed. An occlusal analysis and a full-mouth wax-up on plaster casts were realized. After replacement of old fillings, no-prep PICN restorations (posterior restorations and palatal veneers) were manufactured with respect to the initial wax-up. The restorations (n = 192) were bonded during two consecutive visits within 24 hours. Direct composites were performed to mask the limit between palatal veneers and the anterior tooth buccal face. Patients were treated by a maxillo-facial physiotherapist. Restorations were clinically evaluated after 2 years following FDI criteria. The treatment influence on TMD and patient-centered outcomes following OHIP-49 were assessed.

**Results:** Tooth wear was mainly attributed to sodas consumption and bruxism, and loss of tissue was higher than 50% on most teeth. Mean VDO increase, as estimated with the wax-up, was 5.09 ± 0.85mm on the front teeth. The mean restoration thickness on molars was 0.56 ± 0.21mm and the lowest 0,17 mm. After 2 years, survival rate of restorations was 100% and success rate was 93,75% (11 minor chippings and one debonding). Questionnaires showed a high satisfaction rate of patients with the procedure, the absence of provisional restorations, the one-step VDO increase, the functional and esthetic results and the restorative material. The treatment showed a positive effect on pain (tooth, TMD, neck and back pain) as on the psychological well-being.

**Conclusions:** The One-step No-prep approach is particularly straightforward, minimally invasive, cost-effective and highly appreciated by patients. Present results need to be confirmed by further research.

### Wettability of modified experimental dental adhesives on dentin

R. G. Palma-Dibb[a,b], D. C. De Morais[a], C. Dietrich[a], R. M. Carvalho[a] and A. P. Manso[c]

[a]Department of Oral Biological & Medical Sciences, Faculty of Dentistry, The University of British Columbia, Vancouver, Canada; [b]Department of Operative Dentistry, School of Dentistry, University of São Paulo, Ribeirão Preto, Brazil; [c]Department of Oral Health Sciences, Faculty of Dentistry, The University of British Columbia, Vancouver, Canada

**Introduction:** Compounds as cellulose nanocrystals (CNC) and chitosan (CS) have been used to modify existing dental materials aiming to enhance its mechanical, adhesive and antibacterial properties. However, how they can affect material's wettability it is still unknown.

**Purpose:** To test the effects of silanated and non-silanated compounds (CNC and CS) on the wettability of experimental dental adhesives.

**Materials and Methods:** Resin blends (50%BisEMA; 30%TeEGDMA; 14%HEMA; 4%ethanol; 2%photoinitiators) were added to CNC or CS, silanated(-S) or non-silanated(-NS) at 0.5%, 1.0%, 1.5% or 3.0% by weight (n = 10). Flat dentin surfaces with standardized smear layer were treated with 35% phosphoric acid (15") and rinsed. Moist dentin surface was positioned in Goniometer (Rame-Hart Inc) and 30µmL of experimental adhesive was dropped. Contact angle was measured for 1min. Data were analyzed by one-way and two-way ANOVA, and Tukey test ($p < .05$).

**Results:** Overall, CNC-NS and CNC-S were statistically similar to control ($p > .05$), except for 3%CNC-S with the lowest contact angle ($p < .05$). In contrast, CS-NS showed statistically higher contact angle than CS-S ($p < .05$) overall, while both were similar to the control ($p > .05$). Only for 3% concentrations a significantly lower contact angle was observed for the silanated compounds, CNC-S and CS-S ($p < .05$).

**Conclusions:** Resin blends modified by CNC and CS did not present significant changes in wettability to dentin compared to the control. The 3%CNC-S and 3%CS-S were the most favourable concentration for the experimental resin blend and they can be considered as alternatives for modifications on dental adhesive resin blends without significant changes of wettability in dentin.

**Table 1.** Contact angle (ɵ) measurement (mean ± SD).

| | 0.5% | 1.0% | 1.5% | 3.0% | All Groups |
|---|---|---|---|---|---|
| CNC-NS | 23.4 ± 7.4aA | 19.3 ± 7.0aA | 20.2 ± 6.1aA | 19.9 ± 5.5bA | 20.7 ± 6.5a |
| CNC-S | 25.4 ± 5.5aC | 23.4 ± 6aBC | 17.1 ± 4.8aAB | 13.5 ± 2.0aA | 19.8 ± 7.9a |
| Control | | | | | 17.8 ± 7.4a |
| CS-NS | 20.5 ± 4.6aA | 19.9 ± 8.0aA | 20.7 ± 7.8 aA | 23.6 ± 7.4bA | 21.2 ± 7.0b |
| CS-S | 16.1 ± 2.8aA | 14.8 ± 4.0aA | 19.2 ± 7.8 aA | 16.2 ± 2.9aA | 16.5 ± 4.9a |
| Control | | | | | 17.8 ± 7.4ab |

Lower cases letters compare treatments and capital letters compare concentrations.

### Acknowledgements

Start-up funds (Carvalho RM) and start-up funds (Manso AP) at UBC Faculty of Dentistry; fellowship award (Palma-Dibb RG) at São Paulo Research Foundation, FAPESP, Brazil, (process n. 2017/19229-9).

### Inhibition of artificial carious lesion by “bioactive” resin composites

D. Papadogiannis, M. Dimitriadi and G. Eliades

Department of Biomaterials, School of Dentistry, National and Kapodistrian University of Athens, Greece

**Purpose:** To evaluate the in vitro anticariogenic capacity of recently introduced bioactive restorative materials.

**Materials and Methods:** The bioactive restorative materials tested were Activa (Pulpdent/AC) and Cention (Ivoclar-Vivadent/CN). A glass-ionomer (Equia-Fil, GC Int/GI) and a resin composite (G-aenial Universal Flo, GC Int/RC) were used as positive and negative controls. Sound premolars extracted for orthodontic reasons were randomly classified into 4 groups (n = 6). Standardized class I cavities were prepared in all teeth (Ø=1.5 mm, h = 2 mm) and restored with each material. AC, CN and GI were placed directly into the cavities, whereas for RC, selective acid-etching (37% gel) and adhesive pretreatments were performed (Prime& Bond One, Sirona Densply). After finishing, polishing and tooth isolation with a nail varnish, the specimens were immersed in an artificial caries gel (80 mM lactic with, pH 4.5) for 3 days, water rinsed and then transferred to an artificial saliva solution for 2 days. This sequence was repeated in triplicate. Then longitudinally thin sections (100 μm) were prepared from each specimen, employing a target surfacing system. The presence of artificial lesions (length and depth measurements) adjacent to restorative margins was examined by transmission polarized-light microscopy. Statistical analysis was performed by ANOVA plus Tukey test ($\alpha = 0.05$)

**Results:** Lesions were observed in all specimens, with significantly greater length than width. The ranking of the lesion lengths were RC>AC>CN>GI, whereas for lesion widths AC>CN, RC>GI ($p < .05$).

**Conclusion:** The better performance of GI is probably associated with the molecular bonding to dental hard tissues and release of species with anticaries effect (F, Sr, Ca). The two resin composite bioactive materials, regardless the claimed Ca release, do not seem to offer advantages in lesion length, when not bonded to the cavity walls. From the two bioactive materials, CN resulted in less extended lesions.

### Hydraulic calcium-silicate cements express Ca/Si-release and pH-related antimicrobial capacity

M. S. Pedano, X. Li, I. Nedeljkovic, K. Yoshihara, K. Van Landuyt and B. Van Meerbeek

**Purpose:** To evaluate the antibacterial capacity of the freshly mixed hydraulic calcium-silicate cements (‘hCSCs’) Nex-Cement MTA (‘Nex-MTA’; GC) and Biodentine (Septodont), and the resin-based hCSC TheraCal LC (Bisco), versus that of set cements, this by means of turbidity and colony-forming unit (CFU) assays (n = 3 per experimental group/condition). pH and Ca/Si release were also measured.

**Materials and Methods:** 1.5-cm$^3$ hCSC disks, placed in 24-well plates under sterile conditions, were allowed to set at 37 °C/5%$CO_2$ for 24h. In addition to set hCSCs, freshly mixed hCSCs were immediately exposed to 1.5-ml brain heart infusion (BHI) per sample. Eluates from both conditions were collected after 24h and inoculated with *S. mutans* in 96-well plates, achieving a final bacterial concentration of $2 \times 10^7$/ml. At 3h, 6h, 9h and 24h of incubation (37 °C/5%$CO_2$), turbidity of cultures was measured spectrophotometrically (Varioskan, Thermo-Fisher). Fresh BHI, glucose-supplemented BHI, antibiotics-supplemented BHI and sterile material eluates served as controls. Subsequently, blood-agar plates were inoculated with 50 μl dilutions of the 24h cultures; after 48h of incubation (37 °C/5%$CO_2$), CFU count was determined. For pH and Ca/Si release, the same setting was used as for the antibacterial assay but using sterile-deionized water instead of BHI. Ca/Si release and pH were measured with ICP-OES (Varian 720-ES, Agilent) and with a pH meter (3210 WTW, equipped with a Hamilton MiniTrode electrode), respectively, both at 1h, 24h, 72h, 1w, 2w and 4w.

**Results:** Freshly-mixed hCSCs exhibited antibacterial activity against *S. mutans* following both the turbidity and CFU assays, in this order: Biodentine > Nex-MTA > TheraCal LC ($p < .05$). Freshly mixed cements were more antimicrobial than set hCSCs ($p < .05$), corresponding to a higher pH and higher Ca/Si release from the freshly mixed than from the set hCSCs ($p < .05$).

**Conclusion:** Hydraulic calcium-silicate cements revealed antimicrobial activity against *S. mutans*, in relation with their Ca/Si release and alkaline pH.

### Effect of silane treatment conditions on flexural strength of FRC poles

T. Peltola[a], L. V. Lassila[b] and E. Säilynoja[a,b]

[a]Stick Tech Ltd., Finland; [b]Institute of Dentistry and Turku Clinical Biomaterials Centre – TCBC, University of Turku, Finland

**Purpose:** Unidirectional fibre-reinforced composites (FRCs) can be used as raw material for various dental application, like root canal posts or CAD-CAM materials. To achieve optimum wettability with resin and mechanical properties of FRC an appropriate surface treatment of fibres needs to carry out. The surface treatment depends on the type of fibres and resin used. The purpose of this study was to evaluate the effect of silane treatment conditions on flexural strength and visual homogeneity of the FRCs made by using glass fibres and methacrylate-based resin.

**Materials and Methods:** 3-(Trimethoxysilyl)propyl methacrylate (MPS) treatment was to use for silanization of the glass fibers. The about 15 min MPS treatment was carried out at vacuum, ambient pressure or ultrasonic bath. The post-treatment times and temperatures to stabilize the surface layer were 4, 6 as well as 20 hours and 60, 80 and 100 °C. The flexural strength of the test specimens (n = 9) were measured using universal mechanical testing machine (Lloyd lrx) using loading speed 5.0 mm/min. The length of the test specimen was 65 mm (50 mm span length) and diameter 2.7 mm. The homogeneity of impregnation was evaluated visually from cross-sectional images using light microscope. Statistical analysis was carried using ANOVA ($p < 0.05$).

**Results:** Different MPS-treatment conditions reveal statistically significantly different strength values ($p < 0.05$). The strongest FRC were obtained if the MPS treatment of the fibres was carried out at ultrasonic bath. The treatment conditions had significant effect on the flexural strength of the FRC. 4 hours at 100 °C post-treatment conditions were the best in terms of stabilizing the hydrolysis reaction between silane molecules and OH-groups at glass surface.

**Conclusion:** The best mechanical properties were obtained when fibres were treated in ultrasonic bath. In addition, it can be concluded that short post-treatment time (4 hours) at high temperature (100 °C) eventually revived the strongest FRC.

### Surface characteristics and color stability of gingival-colored resin composites

A. Petropoulou, M. Dimitriadi, A. Sarafianou, S. Zinelis and G. Eliades

Departments of Prosthodontics and Biomaterials, School of Dentistry, National and Kapodistrian University of Athens

**Purpose:** To investigate the surface characteristics and color stability of gingival-colored composite restorative materials simulatıng soft-tissue in implant retained restorations.

**Materials and Methods:** The materials tested were Anaxgum (AN/Anaxdent), Ceramage (CM/Shofu) and Gradia Gum (GG/GC Int). Disk-shaped specimens (Ø=8 mm,h = 2mm) were prepared from each material, cured (5 min) and polished according to the manufacturers' instructions. The microstructure, surface composition and degree of cure (DC%) were examined by BE-SEM/XEDS (n = 3) and ATR-FTIR (n = 6). Roughness parameters were measured by optical profilometry (Sa,Sz,Sdr,Sc n = 6) and hardness by a Vickers indenter (n = 6). For the color stability, specimens were divided into three groups (n = 12/group). Colorimetric (L*,a*,b*) measurements were taken at baseline and following 30d immersion in distilled water, coffee, red wine, and the color differences (ΔE) were calculated. VH measurements were taken again on immersed specimens. Statistical analysis was performed by one-way ANOVA (DC%), Kruskal-Wallis (roughness, VHN) and two-way ANOVA (color difference), plus Tukey post-hoc tests (a = 0.05).

**Results:** AN, GG contain prepolymerized particles (resin/silica) in an aromatic and aliphatic resin matrix respectively, whereas CM contains inorganic zirconia/silica particles in an aromatic resin matrix, with smaller particle size and higher filled surface-area fraction. The DC% ranged between 50.4-55% ($p > .05$). There was no statistically significant difference in the roughness parameters among the materials, except of Sdr in CM (greatest, $p < .05$). AN showed ΔE>3.3 after immersion in all media, CM in coffee and wine and GG in coffee. All changes were associated with increase in b* (yellow shift). Coffee and wine produced the greatest ΔE in CM (11.4 and 7.5 respectively). CM was the hardest material but it was the only one demonstrating a significant reduction in VHN after wine storage

**Conclusion:** Although the inorganic-filled material was the hardest, it demonstrated increased Sdr roughness, the highest ΔE changes (coffee, wine) and the greatest hardness reduction after wine storage.

### Effect of the supporting direction of 3D printing on mechanical properties of pure Ti and Ti alloy

L. X. Ping[a], P. E. Ling[b], G. Hui[a], W. Y. Xin[a] and M. X. Feng[a]

[a]Nanjing Stomatological Hospital, Nanjing University, Nanjing, China; [b]Nanjing QianZhi Technology Company, Nanjing, China

Pure titanium as denture frameworks materials has good compatibility, resistance erosion and high specific strength, Recently, a novel additive manufacturing (AM) technology was used in dental field to fabricate the denture metal framework, such as the selective laser sintering, selective laser melting, and electron beam melting. However, there is not reported to print the pure titanium framework by AM procedures.

**Materials and Methods:** 40 tensile test specimens (n = 10/group) were printed with the vertical and horizontal supporting direction respectively by SLM. Power alloys used were Ti6Al4V (TC4) and pure titanium (TA2), the particle size is about 15 ~ 50 μm. their mechanical properties were evaluated by using a test for tensile strength. A standard maxillary first molar (resin tooth) with a deep chamfer margin was scanned and then to be milled 20 metal dies. 20 crowns (n = 10) were printed by TC4 and TA2 respectively. Marginal discrepancies were measured using an optical microscope at ×100 magnification. SEM was used to check the tissue structure of the printing TA2 and TC4

**Results:** Table 1 shows the effect of the supporting direction on mechanical properties of TA2 and TC4. The supporting direction of 3D printing affected the mechanical properties of the final objects using TA2 or TC4. The strength and elastic modulus of TA2 and TC4 printed with the vertical supporting direction (VP) were lower than the vertical supporting direction (HP), but the elongation rate is higher. The marginal space was 65.6 + 12.6μm for TA2, 75.3 + 14.3 μm for TC4, there is no significant difference. SEM indicated there were many voids in the specimens

**Conclusion:** The titanium framework made using SLM technology showed good mechanical properties and dimensional precision. AM technology is becoming an alternative to subtractive manufacturing or traditional casting technology since it produces little material waste, reproducible manufacture and also is energy efficient.

**Table 1.**

| Metal | N | Elastic Modulus GPa | Yield Strength MPa | Tensile Strength MPa | Elongation A% |
|---|---|---|---|---|---|
| TA2VP | 10 | 105.8 ± 23.8 | 438.9 ± 5.6 | 522.8 ± 4.8 | 32.9 ± 4.1 |
| TA2HP | 10 | 116.1 ± 15.2 | 447.4 ± 9.9 | 553.3 ± 8.1 | 25.3 ± 4.7 |
| TC4VP | 10 | 121.7 ± 15.5 | 1021.7 ± 24.3 | 1088.7 ± 13.3 | 8.5 ± 1.3 |
| TC4HP | 10 | 125.8 ± 12.3 | 1102.1 ± 22.6 | 1167.7 ± 33.5 | 4.4 ± 0.9 |

### Survival rates and deformation of implants with one-piece zirconia crowns

N. C. Ramos[a], A. A. Alonso[a], L. C. Anami[b], R. M. Melo[a], F. R. Oliveira[c], J. C. Dinato[d] and M. A. Bottino[a]

[a]Sao Paulo State University (UNESP), Sao Jose dos Campos, Sao Paulo, Brazil; [b]University of Santo Amaro (UNISA), Santo Amaro, Sao Paulo, Brazil; [c]Conexão Sistemas de Prótese, Aruja, Sao Paulo, Brazil; [d]Federal University of Santa Catarina, Florianopolis, Santa Catarina, Brazil

**Purpose:** The long-term performance of implant connections and crowns depend on the component characteristics and jointing materials. This study aimed to evaluate the survival rates of several external hexagon implants directly connected to zirconia crowns after thermomechanical fatigue. The strain of the hexagons and the integrity of zirconia crowns were also evaluated.

**Materials and Methods:** One-piece zirconia crown ($ZrO_2$), and four different external hexagon dental implants ($n = 9$) (Biotechnology 3i, Conexão, Nobel Biocare, and Neodent) were mounted, and embedded in acrylic resin 30° inclined in relation to the loading applicator. The specimens were submitted to thermomechanical cycling, with $2.5 \times 10^6$ cycles at 3.0 Hz frequency, and 200 N loading. The interface of the implant/zirconia crown system, zirconia crowns quality, and the implant hexagon surface were evaluated under stereomicroscopy and Scanning Electron Microscopy. A nanohardness analysis was performed to verify the hardness of zirconia and implants. Statistical analysis was performed using the Kaplan-Meier test, Multi-Sample Survival Tests, Logrank Test, ($p = 0.05$).

**Results:** The data did not show significant differences in the survival rates of all groups. However, some crowns presented fractures (16.67%) and the external hexagon region of the implants presented plastic deformations (100%). There were no significant differences among the nanohardness of each implant ($p > 0.05$).

**Conclusion:** Within the limitations of the current study, most of the zirconia crowns survived the thermomechanical aging. However, the external hexagon region of implants presented permanent deformation.

### Material thickness and bonding tooth substrate are determinant factors in minimal invasive restorative dentistry

G. T. Rocca, B. Baldrich, C. M. Saratti, M. Delgado, M. Roig, R. Daher and I. Krejci

**Purpose:** To evaluate the influence of restoration thickness and bonding tissue substrate on fracture strength and failure behaviour of minimally invasive CAD-CAM composite resin overlay restorations.

**Materials and Methods:** Eighty simplified bi- and tri-layer cylindrical overlay model including the restoration bonded over bovine tooth dentin (Groups D, $n = 40$) and enamel-dentin (Groups E, $n = 40$) were assembled (diameter 9 mm). Restorations were milled from CAD-CAM composite resin blocks (Brilliant Crios, Coltène/Whaledent AG) in four different thicknesses of 0.5mm, 1mm, 1.5mm, 2mm and equally distributed in four Groups D and four Groups E ($n = 10$). All specimens were submitted to an Hertzian load-to-failure contact test with a spherical contact indenter over a flat-layer structure. Critical loads were recorded in Newton and data were analysed using Kruskal–Wallis test for multiple and Mann-Whitney test for 2-samples comparisons ($p < .05$). Fragments were examined using SEM. The stress distribution for specimens with restorations of 0.5 mm and 2 mm was also investigated with FEA.

**Results:** For both Groups E and D, the mean static loads in Newton increased with an increase in restoration thickness. On contrary, restorations with the same thickness displayed higher resistance values when bonded over enamel than dentin, except for the 2-mm thick restorations. A damage competition was detected between cone/median cracks originating close to the loading contact area of the restorations and radial cracks beginning at their inner surface, with the former prevailing in restorations bonded on enamel and the latter being dominant for restorations bonded on dentin. FEA showed that, in modelled restorations bonded to enamel, maximum principal stresses were concentrated at the top surface of the restoration and into enamel while for restorations bonded to dentin the stress intensity increased nearby the cement layer.

**Conclusions:** For bonded ultra-thin resin composite restorations (0.5 mm to 1.5 mm), enamel as bonding substrate assures higher critical loads to fracture than dentin. This influence gradually decreased as restoration thickened. Conservation of enamel and, therefore, early diagnostic and therapy are crucial when treating eroded/worn posterior teeth with CAD-CAM resin composite overlays.

### How are soluble content and conversion related at different Rp?

R. P. Ruelle[a], E. F. Febrero[a], J. G. Leprince[a,b] and L. D. Randolph[b]

[a]School of Dentistry and Stomatology, Université catholique de Louvain, Brussels, Belgium; [b]Louvain Drug Research Institute, Université catholique de Louvain, Brussels, Belgium

**Purpose:** In dimethacrylate-based dental composites, polymerization rate (Rp) correlates with methacrylate conversion [1]. Increased photoinitiator efficiency was associated to higher Rp, resulting in higher functional group conversion (DC) [2]. Since DC is closely related to monomer addition (versus crosslinking) [3], this work investigates the relationship between DC and soluble content, at different Rp.

**Materials and Methods:** A 70/30 mass% BisGMA/TEGDMA resin was prepared, adding either Lucirin-TPO (TPO) or camphorquinone (CQ) coupled with DMAEMA (CQ:DMAEMA =1:4), at 0.25-0.5-1-2 or 4 mol% of the resin (10 different formulations). Fillers were added: 10mass% of nano silica (AEROSIL R7200, Evonik) and 65mass% micro barium glass (K5 grind, SCHOTT AG). Disks were light cured in polyethylene molds (5mm diameter, 0.5mm thickness), using the AURA light engine (output at 405 nm(TPO) or 470 nm(CQ), irradiance 250-500 or 1000 mW/cm$^2$ and irradiation time ranging from 0.5s to 40s). Raman spectroscopy was used to determine the degree of conversion (n = 3). Disks were placed in ethanol for 168 hours then left to dry for 24h and were then weighed again, to determine the soluble content (n = 3).

**Results:** A positive relationship was observed between photoinitiator concentration (inflexion at 0.5mol%) and DC and a negative one between photoinitiator concentration and soluble content. With increasing DC, differences in soluble content decreased regardless of photopolymerization parameters. Minimal variations were noticed beyond DC = 60%.

**Conclusion:** DC was clearly related to the soluble content, with possible structural differences depending on photopolymerization parameters (different Rp), particularly at low DC (below 60%). A more efficient photoinitiator than CQ was associated to higher DC but there were no clear evidence of a more crosslinked network. This could be detailed by increasing the conversion overlap between CQ and TPO data.

### References

[1] Dickens SH, Stansbury JW, Choi KM, et al. Photopolymerization kinetics of methacrylate dental resins. Macromolecules. 2003;36(16):6043–6053.

[2] Randolph LD, Steinhaus J, Möginger B, et al. Photopolymerization of highly filled dimethacrylate-based composites using Type I or Type II photoinitiators and varying co-monomer ratios. Dental Materials. 2016;32(2):136–148.

[3] Leprince JG, Palin WM, Hadis MA, et al. Progress in dimethacrylate-based dental composite technology and curing efficiency. Dental Materials. 2013;29(2):139–156.

### Effect of preparation taper on fracture resistance of zirconia crowns

C. Schriwer, N. R. Gjerdet and M. Øilo

Department of Clinical Dentistry, Faculty of Medicine, University of Bergen, Norway

**Purpose:** The aim of this study was to evaluate whether the taper of the abutment (convergence angle) affect the fracture resistance or fracture modes of monolithic zirconia crowns.

**Materials and Methods:** A model premolar tooth was prepared with a taper of 15° and a shallow circumferential chamfer preparation. Two additional models were made based on the master model with a taper of 10° and 30°, respectively, using computer design software. Twenty monolithic zirconia crowns (DD CubeX$^2$, Dental Direkt GmbH) were produced for each model (n = 60). The cemented crowns were centrally loaded in the occlusal fossa in a servo hydraulic testing system at 0.5 mm/min until fracture. Fractographic analysis was performed on all fractured crowns.

**Results:** The crowns with 30° taper fractured at lower loads compared to the crowns with a taper of 10° and 15° (Table 1, $p < .05$). For 47/60 crowns the fracture origin was in the cervical area. The fracture started close to the top of the curvature in the mesial or distal crown margin. The remaining fractures started at the internal surface of the occlusal area and propagated to the approximal cervical area on both sides.

**Conclusion:** The fracture resistance of the monolithic zirconia crowns decreased with increasing convergence angle. All fracture modes were similar to fractures observed clinically.

**Table 1.** Median and range for load at fracture for the different test groups (n = 60).

| Convergence angle (degrees) | Median | Range |
|---|---|---|
| 10 | 1870 | 642–2495 |
| 15 | 2015 | 1087–2583 |
| 30 | 1229 | 771–1769 |

### Antibacterial activity of BisGMA/TEGDMA composites – one year evaluation *in vitro*

L. T. Sampaio Silva, L. C. Anami-Paulin, L. P. M. Campo and L. C. C. Boaro

**Purpose:** The aim of this study was to evaluate the antimicrobial activity of hybrid composites containing MMT/CHX complex and conventional filler (barium glass and silica) in different concentrations.

**Materials and Methods:** Five BisGMA/TEGDMA based composites were prepared, containing 5% of montmorillonite (MMT) loaded with chlorhexidine, the total filler amount were 0, 30 or 60% by weight. The proportion of barium glass/colloidal silica was 80/20 or 70/30 by weight. Antimicrobial activity was evaluated through the inhibition halo test, and the bacterium used was *S. Mutans*. For each composite 60 specimens were made. These specimens were stored in distilled water at 37 °C, and the water was exchanged weekly. Each month, 5 specimens were positioned on the surface of agar plate containing the *S. Mutans* and incubated in anaerobiosis. The inhibition halo was measured in mm using a digital caliper. Data were analyzed using Kruskal-Wallis/Dunn and the level of significance adopted was 5%.

**Results:** Selected results are shown in table:

**Conclusion:** Within the limitations of this study we can conclude that a greater amount of inorganic filler promoted a more controlled release of chlorhexidine, represented by the formation of halo of inhibition in prolonged periods.

**Table.** Mean (SD) in mm for inhibition halo through the one year evaluation at selected months. In the same columm, values followed by the same letter are statistically similar ($p > .05$).

| Barium glass/silica (wt.) | | Month | | | | |
|---|---|---|---|---|---|---|
| Ratio | Amount | 1 | 2 | 3 | 6 | 12 |
| 80/20 | 60% | 6,3 (1,3)$^{AB}$ | 4,3(1,3) | 1,6(0,6)$^{AB}$ | 1,3(0,4)$^{AB}$ | 0,0(0,0)$^{C}$ |
| | 30% | 5,9 (1,7)$^{AB}$ | 4,5(1,2) | 1,6(0,5)$^{AB}$ | 5,2(1,7)$^{A}$ | 0,0(0,0)$^{C}$ |
| 70/30 | 60% | 12,4 (2,5)$^{A}$ | 4,6(0,8) | 2,1(0,2)$^{A}$ | 2,5(0,8)$^{AB}$ | 2,8(1,0)$^{B}$ |
| | 30% | 10,1 (1,7)$^{A}$ | 3,2(0,4) | 1,6(0,5)$^{AB}$ | 0,0(0,0)$^{B}$ | 0,0(0,0)$^{C}$ |
| Control | | 3,8 (1,0)$^{B}$ | 6,8(1,4) | 0,0(0,0)$^{B}$ | 0,0(0,0)$^{B}$ | 6,9(0,3)$^{A}$ |

### Effect of position and quantity of micro retentive groove for bond strength of composite block

A. Shinya[a,b], A. Niitsuma[a], S. Shiratori[a], S. Katsunuma[a] and H. Gomi[a]

[a]The Nippon Dental University, Tokyo, Japan; [b]University of Turku, Turku, Finland

**Purpose:** To improve the total bond strength, we especially focused on the mechanical interlocking for CAD/CAM fixed prosthesis. We designed new milling program for crown inner surface based on the CAD/CAM technology, and it was possible to shape the deliberate groove (Micro Retentive Groove: MRG). The purpose of this study was to evaluate the effect of MRG position and quantity on the pull-out bond strength of a self-adhesive resin cement to CAD/CAM resin composite crown.

**Materials and Methods:** Standard master crown model was designed with height of 3.5mm, diameter of 6.0mm, 1.0mm-round marginal finish line, and 6 degrees taper. MRG was designed with 100μm depth. To evaluate the position of MRG, five different position of MRG: 1.375mm (P1) lower side from crown inner top surface, 1.750mm (P2), 2.125mm (P3), 2.500mm (P4), and 2.875mm (P5) were tested. For the effect of MRG quantity, three different numbers of MRG: one MRG milled at the P3 position (Q1), three MRGs at the P2, 3 and 4 (Q3), five MRGs (Q5), and no MRG (Q0, control) were tested. All fabricated crowns (CERASMART270, GC) were cemented with self-adhesive resin composite cement (G-CEM ONE, GC). After water storage at 37 °C for 24 h, the pull-out bond strength test was carried out. Specimens were loaded until failure using a universal testing machine at a crosshead speed of 1.0mm/min, and the results were analyzed by one-way ANOVA ($p < .05$).

**Results:** The highest pull-out bond strength of different position was measured at P3 (10.5 ± 1.3 MPa), but no significant differences were shown. In difference of quantity, the highest value was measured at Q3 (11.2 ± 1.6 MPa), and significant differences were observed between Q3 and Q0, Q5.

**Conclusion:** The best position and quantity of MRG is one or three MRGs at the middle of height of the crown.

### Fracture resistance of high and super translucent zirconia monolithic crowns

A. Skjold, J. A. Indergård, H. Gjengedal, N. R. Gjerdet and M. Øilo

Department of Clinical Dentistry, Faculty of Medicine, University of Bergen, Norway

**Purpose:** Evaluate the effect of thermal and mechanical aging of monolithic zirconia crowns with different material composition. The super translucent zirconia (5Y-TZP) has a higher content of cubic crystals than the high translucent zirconia (3Y-TZP).

**Materials and Methods:** Sixty crowns made on a shallow chamfer premolar preparation, thirty super translucent crowns (DDcubeX[2], Dental Direkt GmbH) and thirty high translucent crowns (DDBioZX[2], Dental Direkt GmbH) were cemented with self-adhesive resin cement (RelyX Unicem, 3M ESPE) on composite stubs (SDR, Denstply Sirona Restorative). A group of ten crowns from each material were subjected to dynamic loading (200N at 1 Hz, 30000 cycles) in water at 37 °C. A second group of crowns (n = 10) were subjected to hydro-thermal exposure for 3 × 20 min at 134 Celsius 3.2. Third group of crowns (n = 10) were left untreated as controls. All crowns were subjected to axial loading in a servo hydraulic testing system at 0.5 mm min$^{-1}$ until fracture occurred. The results were statistically analyzed with non-parametric tests.

**Results:** The super translucent crowns had median load at fracture at 1938N (1283N-2723N). The high translucent crowns had median value at 3449N (3021N-5112N). There were no significant differences in fracture load among the three groups within each material type. All three groups of high translucent crowns had significantly higher fracture values than the super translucent crowns ($p < .005$).

**Conclusion:** The high translucent crowns fractured at higher loads than the super translucent crowns. The mechanical and hydro-thermal aging procedures did not affect the values for any of the crowns.

### Core build-up materials: a comparative study

L. Spinhayer[a], A. Bui[a], J. G. Leprince[a,b] and C. M. F Hardy[a,b]

[a]School of Dentistry and Stomatology, Université catholique de Louvain, Brussels, Belgium; [b]Louvain Drug Research Institute, Université catholique de Louvain, Brussels, Belgium

**Purpose:** It is common, under a peripherical crown to build a direct resin composite (RC) core. The impact of the self and light curing effect in depth in this core is a frequent question. Some articles analysed the monomers conversions in depth of this kind of RC [1], others conclude that the performances of the direct materials chosen depend on their formulation, as well as on the respective curing process [2]. The purpose of this study is to compare the physico-mechanical properties of a selection of core build-up RC available on the market with conventional light-cure RC, after being light cured or self-cured only.

**Materials and Methods:** Rectangular specimens of core build-up composite resins were prepared using a 25x2x2mm Teflon mould (based on ISO 4049 specifications). In the control group, the specimens were light cured during 20s in three consecutive spots (n = 20) with the Bluephase G2 (Ivoclar Vivadent®). For the chemo polymerisation-only, the material was covered by an aluminium foil during 10 minutes to avoid photopolymerization by the natural light. After being cured, the specimens were carefully removed from their mould. Each specimen was polished and calibrated, before being stored in distilled water at 37 °C for one week. The elastic modulus and flexural strength were measured using a three-point bend test at a strain rate of 0,75mm/min until fracture (machine Instron 5566, High Wycombe, UK).

**Results:** The preliminary results show no significant difference between the results of the light and the self-cure samples. The Young Modulus of each RC tested in this study is lower than conventional composite resin tested. Concerning the flexural strength, the tendency shows higher values for the light-cured RC, but the differences are not significant for all RC.
**Conclusion:** That means that polymerization in depth, with weak or no light transmission does not affect significantly the mechanical properties of the RC tested. We would like to point out that the Young Modulus of each RC tested in this study is lower than conventional composite resin tested and is below the dentine's values. The question of the relevance of this type of restoration can be asked because of the weak mechanical properties of a material having to support a crown.

### References

[1] Kournetas N, Tzoutzas I, Eliades G. Monomer conversion in dual-cured core buildup materials. Oper Dent. 2011;36(1):92–97.
[2] Ruttermann S, Alberts I, Raab WH, et al. Physical properties of self-, dual-, and light-cured direct core materials. Clinical Oral Investigations. 2011;15(4):597–603.

### A comparison of Norwegian and overseas made dental metallic crowns

M. Syverud, E. K. Austrheim, A. Mulic and H. Valen

Nordic Institute of Dental Materials (NIOM), Oslo, Norway

**Purpose:** To investigate the composition and conformity with the stated declaration for alloys used for dental crowns among Norwegian and overseas laboratories.
**Materials and Methods:** Tooth 16 of a patient given his informed consent was prepared for single porcelain fused to metal crown. An impression of the upper jaw including the prepared tooth, antagonist impression and a bite registration was performed. This was used to make an epoxy model for production of identical impressions that were distributed to dental clinics ($n = 23$) covering the whole of Norway. The dentist distributed the received impressions and the bite registration to their commonly used laboratory (some in Norway and some overseas) ($n = 38$). The dental laboratory was not informed that the crown would be used for research purposes. The dentist sent the produced crown with the all enclosed information to NIOM for examination. This examination consisted of registration whether a Declaration of Conformity was enclosed or not, analysis of the chemical composition and if the alloys contained toxic elements (Ni, Cd, Be, Pb) above the limit values specified in ISO 22674:2016. These analyses were performed at Sheffield Analytical Services (UK).
**Results:** Analyses of the alloy part of the crowns did not match to the stated composition for 14% of the Norwegian and 8% of the overseas works. However, the composition was not stated for a third of the crowns. The Declaration of Conformity was missing for 29% of the Norwegian and for all of the imported work. Concerning toxic elements, 18% of the works appeared to contain Pb above the permitted limit (0,02 wt %) according to ISO 22674:2016.
**Conclusion:** The study revealed major deficiencies for both Norwegian and overseas made crowns regarding conformity between composition and the stated declaration.

### Resin cements effects and thickness under lithium disilicate structural reliability

I. V. Tanaka, A. F. Nunes Reis, P. H. O. Prado, L. F. Valandro and R. M. Melo

**Purpose:** The aim of this study was to evaluate the effect of the resin cement on the flexural strength and also the structural reliability of an ultra-thin glass-ceramic.
**Materials and Methods:** 120 discs (12 mm diameter) of lithium disilicate (LIDi) (IPS e.max CAD): 60 discs of 0.3 mm and 60 of 0.5 mm thickness were produced. After polishing and crystallization, 40 discs were polished and 80 had surfaces conditioned with 5% hydrofluoric acid for 20 s and silanized. The treated discs were divided into three groups ($n = 20$) according to the cement (Variolink Veneer-V or Panavia F-P). The elasticity modulus (E) of the cements were measured with impulse excitation technique ($V = 8.33 \pm 0.90$ and $P = 10.19 \pm 0.63$ GPa). After cementation ($\sim$100 μm), the samples were stored (water, 37° C/24h) and subjected to biaxial bending, with the cement on the tensile side. Equations for monolithic and bilayered samples were used for stress calculation. For the bilayers, the stress at the interface and at the lower level of the disks were calculated. The data were analyzed using Weibull's method (95% CI) and the failure mode was assessed with scanning electron microscopy.
**Results:** The strength of the non-cemented discs was higher than those of the cemented discs, which did not differ from each other. The E of the cemented discs was larger than the discs without cement. The strength of the cement P was greater than that of V. There were no differences between the moduli of the cement. The fracture origins were on the cementation surface.
**Conclusion:** The cements showed similar E that resulted in similar ceramic mechanical behavior, which not affect the LiDi strength. There was increased structural reliability in both ceramic thicknesses.

### Influence of a novel surface treatment on composite zirconia bonding

S. Teerakanok and R. Giordano

Department of Restorative, Sciences and Biomaterials, Henry M. Goldman School of Dental Medicine, University, Boston, Massachusetts, United States

**Purpose:** To determine the effects of two novel zirconia surface treatments on composite resin bonding to zirconia.
**Materials and Methods:** Eighteen cylindrical zirconia discs were fabricated by die pressing TZ-3YB-E TOSOH (#Z305653B, Yamaguchi, Japan) zirconia powder into cylinders. The cylinders were sectioned into discs and sintered to full density 1300 °C for 6 hours. The specimens were randomly assigned to 2 groups as follows: air-abrasion (AA) and solution coating (SC). AA group was polished, then air-abraded with 50 μm alumina particles at 2 bar for 20s. The SC group was painted with solution containing zirconia, water, porogen, and dispersant. After being dried, the coated discs were packed into a heat-sealed plastic bag, pressed, and sintered at 1300 °C for 6 hours. Both groups were cleaned in an ultrasonic bath of ethanol and distilled water for 10 minutes each. Ceramic primer plus (Kuraraya, Japan) was applied to the air dried zirconia discs. After placing the disc into a holder, clear resin cement (Panavia™ V5 (Kuraraya, Japan) was dispensed into a 4 mm mold diameter centered on the disc. The cement was cured with light (Bluephase Light cure 20i, Ivoclar Vivadent Inc., Schaan, Liechtenstein). Shear bond strength was tested with semicircular blade while surface roughness was tested with Profilometer/SJ-201 (Mitutoyo, Japan). Statistical analysis involved independent T-test using IBM SPSS ($p < .05$).
**Results:** The SC groups recorded a significant higher shear bond strength than the AA group ($P < 0.001$) (SC = 12.02 ± 2.38 MPa and AA = 7.69 ± 2.16 MPa). Moreover, the surface roughness of SC group was also significantly greater than the AA group ($P < 0.000$) (SC = 3.01 ± 0.46 μm and AA = 0.44 ± 0.02 μm).
**Conclusions:** The bond strength and surface roughness of a novel treatment were significantly greater than a conventional treatment.

### R-curve behavior of monolithic and bilayered short-fiber reinforced flowable composites

J. Tiu, R. Belli and U. Lohbauer
Friedrich-Alexander Universität Erlangen-Nürnberg (FAU)

**Purpose:** To characterize the toughening mechanisms and fracture behavior of a conventional flow resin composite and a fiber-reinforced flow resin composite (FRF), as monolithic or bilayered constructs. The orientation of the fibers in the FRF in the R-curve shape was evaluated.
**Materials and Methods:** A conventional resin composite (CF, Essentia HiFlo, GC) and a short fiber-reinforced flowable resin composite (FRF, StickTech GC) were prepared as monolithic or bilayer specimens, either with fibers aligned or randomly oriented. The material(s) were prepared in a mold (2.5 × 5 × 25mm), notched (depth 0.6-0.8mm), polished, and tested (3-point bending according to the single-edge-V-notched-beam technique). As the crack grew, the force/displacement curve reached a maximum and changed compliance. The test was stopped, force recorded, and associated crack length was recorded under a light microscope. Approximately eight values were recorded for each specimen. Stress intensity ($K_{I,appl}$) values were calculated according to ASTM-E1820 and plotted against crack extension to determine the R-curves.
**Results:** In the bilayer system, the crack grew from the precrack through the CF material to the interface after a maximum load. The interface was a toughening point as the crack stalled for both fiber orientations. Within the FRF, the specimens with aligned fibers showed a linear increase in toughness ($K_I$) from ~2 MPa.m$^{0.5}$ up to ~4 MPa.m$^{0.5}$. Conversely, R-curves from specimens with randomly distributed fibers showed a rise in the first hundreds of microns followed by a decline thereafter. Monolithic FRF showed similar toughening behavior to the FRF in the bilayer.
**Conclusion:** The fibers in the FRF material are able to stall propagating cracks whether the material is used by itself or in a bilayer. However, it is most effective when the fibers are aligned perpendicular to the crack front showing a rising R-curve behavior.

### The effect of bacteria adhesion on zirconia stability

J. K. H. Tsoi, A. Han, Y. Yang and J. P. Matinlinna
Faculty of Dentistry, The University of Hong Kong, Sai Ying Pun, HONG KONG

**Purpose:** The current study was to investigate the effect of bacteria adhesion on the chemical and mechanical stabilities of zirconia.
**Materials and Methods:** Eighty of polished and sintered zirconia discs (IPS e.max®ZirCAD MT A2, Ivoclar, Liechtenstein) in round shape (20mm in diameter) were prepared. Porphyromonas gingivalis (*P.g.*) was inoculated on the zirconia discs. Specimens without bacteria incubation were served as control group. Inductive coupled plasma-optical emission spectroscopy (ICP-OES) test was applied to quantify the concentration of ion/particulate species with zirconium element in the medium with and without *P.g.* bacteria incubation. The surface characteristics of zirconia were evaluated by water contact angle (WCA), biaxial flexural strength (BFS) test and X-ray diffraction (XRD) measurements. The statistical analysis was performed with ANOVA with $\alpha = 0.05$.
**Results:** The ion/particulate species with zirconium element detected by ICP-OES in the groups of zirconia incubated in *P.g.* broth with *P.g.* bacteria for 3d (12.38 ± 7.71 ppb) and 7d (11.30 ± 9.43 ppb) were significantly higher than those groups of zirconia incubated in *P.g.* broth without *P.g.* bacteria for 3d (1.12 ± 0.31 ppb) and 7d (1.21 ± 0.39 ppb). WCA of zirconia surfaces after incubated in *P.g.* broth with *P.g.* bacteria for 3d (12.04°±2.05°) and 7d (15.09°±2.95°) were higher than zirconia surfaces after immersed in pure *P.g.* broth without *P.g.* bacteria for 3d (7.17° ± 1.09°) and 7d (7.55° ± 0.65°), however, they were significantly lower than those on the zirconia surfaces without any treatment in the control group (73.46° ± 8.57°). The BFS values of the zirconia after incubated with *P.g.* bacteria for 3d (632.57 ± 119.96 MPa) and 7d (656.17 ± 100.29 MPa) were significantly lower than zirconia without any bacteria incubation (765.01 ± 20.12 MPa). XRD showed no tetragonal (T) to monoclinic (M) phase change of zirconia in all the groups.
**Conclusion:** *P.g.* adhesion on zirconia could change the chemical and mechanical stabilities of dental zirconia and lead to zirconia degradation

### Comparison of physical properties of flowable and conventional resin composites

A. Tsujimoto[a,b], K. Nojiri[b], W. W. Barkmeier[a], R. L. Erickson[a], T. Takamizawa[b], M. Miyazaki[b] and M. A. Latta[a]
[a]Department of General Dentistry, Creighton University School of Dentistry, Omaha, Nebraska, USA; [b]Department of Operative Dentistry, Nihon University School of Dentistry, Tokyo, Japan

**Purpose:** The purpose of this study was to compare flowable and conventional resin composites in terms of physical properties.

**Materials and Methods:** Eleven flowable resin composites (Admira Flow, Beautifil Flow Plus X, G-ænial Flow, G-ænial Flow Universal, Gradio Flow, Herculite Ultra Flow, Parafique Universal Flow, Tetric EvoFlow, Venus Diamond Flow, Venus Flow and X-flow) and five conventional resin composites (Ceram X Universal, Estelite Σ quick, Filteck Surprem XTE, Tetric EvoCeram and Venus Diamond) were used in this study. Flexural strength (FS), flexural modulus (FM), compressive strength (CS), Vickers hardness, volumetric shrinkage and cuspal deflection were determined.
**Results:** The flexural strength, flexural modulus and compressive strength (FS: 102.4–157.4 MPa; FM: 5.0–10.8 GPa; CS: 314.2–479.4 MPa) of flowable resin composites were similar to those (FS: 89.9–167.7 MPa, FM: 7.8–13.1 GPa; CS: 342.2–416.4 MPa) of conventional resin composites. On the other hand, the Vickers hardness of flowable resin composites (18.2–46.4 HV) tended to be lower than that of conventional resin composites (41.2–75.4 HV). The volumetric shrinkage of flowable resin composites (3.7–6.6%) was significantly higher than that of conventional resin composites (2.5–2.9%). The cuspal deflection of flowable resin composites (10.8–29.1 μm) was similar to that of conventional resin composites (13.1–25.1 μm).
**Conclusions:** The flexural properties, compressive strength and cuspal deflection of flowable resin composites were comparable to those of conventional resin composites. On the other hand, the Vickers hardness and volumetric shrinkage of flowable resin composites appeared inferior to those of conventional resin composites.

### The importance of the tetragonal phase in partially stabilized zirconia

F. Zhang[a,b], J. Vleugels[b] and B. Van Meerbeek[a]
[a]KU Leuven (University of Leuven), Department of Oral Health Sciences, BIOMAT - Biomaterials Research group & UZ Leuven (University Hospitals Leuven), Belgium; [b]KU Leuven (University of Leuven), Department of Materials Engineering, 3001 Heverlee, Belgium

**Purpose:** Partially stabilized zirconia (PSZ, sometimes called not correctly as 'cubic' zirconia) containing a higher yttria content than the conventional 3 mol% yttria stabilized zirconia (3Y-TZP) are gaining popularity, mainly due to their higher translucency. The importance of the cubic phase (*c*-$ZrO_2$) is usually emphasized, without much attention for the remaining tetragonal phase (*t*-$ZrO_2$). The aim of this work was to compare the properties of two PSZ ceramics with a different microstructure and *t*-$ZrO_2$ crystal structure.
**Materials and Methods:** Two 5 mol% yttria-stabilized PSZ ceramics were prepared by two different processing routes. One grade was made from a powder in which the yttria stabilizer was homogeneously distributed in the zirconia grains (referred to as T5Y), while another grade was made from a 3 and 8 mol% yttria-stabilized zirconia powder mixture, resulting in a bimodal microstructure (referred to as B5Y). X-ray diffraction (XRD) with Rietveld analysis was used to assess the phase composition and their crystal structure. Mechanical properties were assessed by biaxial bending strength (n = 20) with Weibull analysis. Translucency (n = 6) was measured with a spectrophotometer. The aging stability was evaluated by hydrothermal treatment (n = 6) in steam at 134 °C, after which the tetragonal to monoclinic transformation was measured by XRD and the microstructural changes were investigated by scanning electron microscopy (SEM).
**Results:** T5Y and B5Y showed a similar phase composition (about 40 vol% of *t*- and 60 vol% of *c*-$ZrO_2$) but the crystal structure of the *t*-$ZrO_2$ phases were different. The *t*-$ZrO_2$ in T5Y contained a higher yttria content and a lower tetragonality (c/a) of $1.0126 \pm 0.0002$. The crystal structure of the *t*-$ZrO_2$ phase in B5Y was comparable to that in conventional 3Y-TZP with a tetragonality of $1.0153 \pm 0.0002$. As such, B5Y had a significantly lower biaxial strength with lower Weibull modulus (i.e. lower reliability), lower translucency and higher aging-susceptibility compared to T5Y.
**Conclusion:** The microstructure and the crystal structure of the tetragonal phase had a determinant role on the properties of partially stabilized zirconia.

### Fracture surface and clustering behaviour of zirconia reinforced high impact acrylic resin

S. Zidan[a], N. Silikas[a], J. Haider[b] and J. Yates[a]
[a]School of Medical Sciences, Division of Dentistry, University of Manchester, Manchester, United Kingdom; [b]School of Engineering, Manchester Metropolitan University, Manchester, United Kingdom

**Purpose:** The aim of this study was to investigate the fracture toughness, fracture surface and clustering behaviour of particles on the surface of high impact heat-cured denture base acrylic resin impregnated with different concentrations of yttria-stabilized zirconia ($ZrO_2$) nanoparticles.
**Materials and Methods:** Sixty single edge notched specimens were fabricated with high impact heat-cured acrylic resin reinforced with $ZrO_2$ nanoparticles, the dimensions of the specimens were (40 mm × 8 mm × 4 mm). All the specimens were divided into six groups: Group 1 was the control group with no zirconia and other groups were reinforced with different wt% concentrations of $ZrO_2$ nanoparticles (1.5%, 3%, 5%, 7%, and 10% weight respectively). Fracture toughness (a single edge bending test) was carried out for each specimen and critical force (P) and critical stress intensity factor ($K_{IC}$) values recorded. Surface analysis of one specimen in each group was performed with a scanning electron microscope (SEM) to observe the fracture and clustering on a specimen surface. The Kruskal-Wallis test was used to analyse the results and to observe any significant difference ($P < 0.05$).
**Results:** All groups reinforced with $ZrO_2$ showed lower fracture toughness, except the G4 (5 wt%) showed that the mean values of fracture toughness slightly increased ($2.14 \pm 0.1$ MPa) when compared to control group ($2.12 \pm 0.1$ MPa). However, it was not statistically significant ($P > 0.05$). SEM images indicated some voids and clustering increased with $ZrO_2$ at high concentrations.

**Conclusion:** Adding zirconia nanoparticles in concentration of 5wt% to be useful in increasing the fracture toughness of high impact acrylic resin.

### Microstructural and mechanical characterization of high translucent zirconia-based materials

S. Zinelis, X. Barmpagadaki, M. Dimitriadi and G. Eliades

Department of Biomaterials, School of Dentistry, National and Kapodistrian University of Athens, Greece

**Purpose:** To characterize the microstructure and the mechanical properties of a new generation zirconia-based materials providing increased translucency.
**Materials and Methods:** The materials tested are presented in Table 1.

Table 1. Materials and flexural strength (three-point bending test) as provided by the manufacturers.

| Material | Code | Flexural Strength (MPa) |
|---|---|---|
| BruxZir Solid Zirconia Milling Blanks | BZR | 625–675 |
| KATANA Zirconia HT | KHT | 1125 |
| KATANA Zirconia ML | KML | 1125 |
| KATANA Zirconia STML | KST | 748 |
| KATANA Zirconia UTML | KUT | 557 |

Disk-shaped specimens (n = 5) were prepared from each material and Raman spectra were acquired employing a Raman microscope to determine the monoclinic to tetragonal ratio (m/t). The mechanical properties of the materials were determined by Instrumented Indentation Testing (IIT, ISO 14577-2002). The properties tested were Martens hardness (HM), indentation modulus ($E_{IT}$) and elastic index ($\eta_{IT}$). The results of HM, $E_{IT}$ and $\eta_{IT}$ were statistically analyzed employing one-way ANOVA and SNK test ($\alpha = 0.05$).
**Results:** The results are summarized in Table 2. KST showed significantly higher HM and $E_{IT}$ compared to all other materials, which share similar mechanical properties. Insignificant differences were identified for $\eta_{IT}$ denoting that all materials share the same brittleness. KUT showed the highest and KHT the lowest m/t ratio.
**Conclusions:** The materials tested showed differences in microstructure and mechanical properties, which may anticipate differences in their clinical performance.

Table 2. Mechanical properties and m/t ratio of the materials tested (means, sd). Same superscripts show values with statistically insignificant differences ($p > 0.05$).

| Material | HM (N/mm$^2$) | $E_{IT}$ (GPa) | $\eta_{IT}$ (%) | m/t ratio |
|---|---|---|---|---|
| BZR | 7693 (1202)$^1$ | 166 (24)$^1$ | 50 (2)$^1$ | 10.2 (1.8)$^1$ |
| KHT | 8628 (823)$^1$ | 186 (22)$^1$ | 48 (2)$^1$ | 8.4 (2.2)$^1$ |
| KML | 8347 (790)$^1$ | 192 (18)$^1$ | 47 (3)$^1$ | 13.4 (1.4)$^{1,2}$ |
| KST | 10276 (995)$^2$ | 214 (8)$^2$ | 50 (3)$^1$ | 10.4 (1.6)$^1$ |
| KUT | 8295 (1006)$^1$ | 182 (21)$^2$ | 49 (2)$^1$ | 14.2 (0.9)$^2$ |
